# Chromatin Remodeling, DNA Double-Strand Break Repair, and Human Disease: How a Breakup Changes You

**DOI:** 10.3390/biom16040589

**Published:** 2026-04-15

**Authors:** Adriana Chiaramida, Christopher B. Cummings, Thomas L. Clarke

**Affiliations:** Department of Pathology and Laboratory Medicine, Boston University Chobanian and Avedisian School of Medicine, Boston, MA 02118, USA; achiara1@bu.edu (A.C.); chrisbc@bu.edu (C.B.C.)

**Keywords:** DNA damage repair, chromatin, genome stability, histones, post-translational modifications, DNA double-strand breaks

## Abstract

Chromatin architecture is a central determinant of genomic stability. Effective DNA repair requires dynamic chromatin remodeling to grant repair factors timely access to lesions and to orchestrate repair pathway choice. Disruption of chromatin-regulatory mechanisms or DNA damage response pathways undermines repair fidelity and contributes to a wide spectrum of human disorders, including developmental syndromes, premature aging, and multiple cancers. Here, we review how chromatin state and remodeling complexes shape detection, signaling, and resolution of DNA double-strand breaks, and we examine how their misregulation drives disease and presents opportunities for therapeutic intervention. Specifically, we discuss how post-translational modifications and ATP-dependent chromatin remodeling complexes contribute to DNA damage repair with a particular focus on DNA double-strand breaks, one of the most deleterious DNA lesions. We summarize how chromatin remodeling and histone post-translational modifications regulate DNA repair pathway choice, and how these processes are essential for safeguarding genomic integrity and preventing human disease. Finally, we discuss emerging concepts and major unanswered questions in the context of chromatin function and DNA double-strand break repair, with a focus on exploring the emerging literature on the role of chromatin compartments and topological associated domains for orchestrating DNA repair within chromatin and safeguarding genomic stability.

## 1. What Is Chromatin?

**Figure 1:**
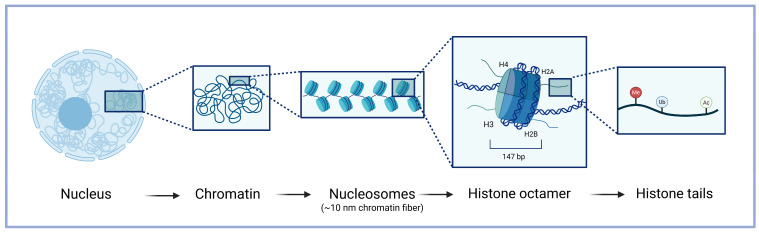
Chromatin  packaging in the nucleus. The nucleus sequesters an enormous amount of genetic material within the cell and provides the architecture for genomic regulation. This is achieved via chromatin, the nucleoprotein filament built from repeating units, known as nucleosomes, that contain 147 base pairs of DNA wrapped around an octameric histone protein complex. Histones contain intrinsic features that mediate epigenetic regulation, most notably their N-terminal tails. These tails are substrates for multiple post-translational modifications, which can alter histone–DNA contacts and create binding sites for effector proteins. All processes in which DNA is the substrate (replication, transcription, and DNA repair) occur in the context of chromatin. Created in BioRender. Chiaramida, A. (2026) https://BioRender.com/e9eld1d.

Chromatin is the dynamic complex of DNA and proteins that packages genetic material within a eukaryotic cell nucleus, enabling the human genome to fit into a confined space while simultaneously regulating gene accessibility (Figure 1). The fundamental unit of chromatin is the repeating nucleosome, which is composed of 147 base pairs of DNA wrapped around an octamer of four core histones (H3, H4, H2A, and H2B); linker histone H1 binds to DNA entry and exit sites and contributes to chromatin compaction [1]. The core histones are predominantly globular, except for their N-terminal tails, which are intrinsically unstructured and highly flexible [2]. These tails serve as key sites for a diverse array of post-translational modifications (PTMs), essential for modulating chromatin structure and function [3]. This, together with direct methylation of DNA bases [4], creates the scaffold for higher-order chromatin organization and plays a crucial role in regulating access to the underlying genetic code, thereby influencing transcriptional activity, DNA replication, and repair processes [5].

Chromatin states, shaped by specific PTMs and histone variants, form distinct structural landscapes that modulate the degree of chromatin compaction and influence DNA accessibility. The majority of chromatin exists as heterochromatin, characterized by densely packed nucleosomes that restrict access to DNA, promoting gene silencing, and maintaining genomic stability [6]. Contrastingly, open or euchromatic regions exhibit reduced nucleosome density or more dynamic nucleosome positioning, which facilitates rapid access for both transcription factors and DNA repair proteins, promoting repair processes alongside active gene expression [6,7]. The dynamic interplay between these repressive and accessible chromatin states governed by nucleosome organization and histone modifications is critical not only for regulating cellular identity and function but also for orchestrating timely DNA repair. Disruptions in this balance can impair DNA repair pathways and are frequently implicated in genomic instability, contributing to the development of cancer and various developmental disorders [8].

### 1.1. Chromatin and DNA Double-Strand Break Repair

#### 1.1.1. Nucleosomes: Protective and Inhibitory

Early models postulated that eukaryotic chromatin served the function of physical packing and storage of the vast amount of DNA in the genome. In this concept, the presence of nucleosomes represents a physical barrier to processes in which DNA is the substrate, such as repair of double-strand breaks (DSBs), limiting accessibility of required repair proteins. Experimental evidence supports this view: nuclease digestion of chromatin results in ~200 base pair fragments through gel electrophoresis, representing sterically protected DNA within the repeat unit of the nucleosome [9]. In the context of repair, nucleotide excision repair (NER) is 10-fold less efficient when nucleosomal DNA is the substrate compared to “naked” DNA [10], and analysis of genome-scale sequence variability shows that single-nucleotide polymorphisms (SNPs) are increased on bulk nucleosomes compared to linker regions of DNA [11].

By contrast, nucleosomes have been shown to be protective against IR-induced damage [12], compact chromatin reduces the propensity of DNA to form adducts with damaging drugs like cisplatin [13], and indels are depleted in nucleosomal DNA [11]. Taken together, these results suggest that nucleosomes play two seemingly antagonistic roles in the context of DNA damage: (1) steric hindrance that limits protein accessibility for repair, and (2) partial attenuation of DNA damage induced by radiation and chemical agents.

#### 1.1.2. DNA Double-Strand Break Repair

Among DNA lesions, DSBs are a genomic emergency: unrepaired DSBs cause cell death, whereas misrepair leads to mutation, chromosomal translocation, and genomic instability [14]. As such, the cell has evolved two main pathways that recognize and respond to DSBs, namely non-homologous end-joining (NHEJ) and homologous recombination (HR). There are also lesser-used mutagenic pathways such as single-strand annealing (SSA), microhomology-mediated end joining (MMEJ), and alternative non-homologous end joining (alt-NHEJ), which are reviewed extensively [15,16,17]. NHEJ requires minimal DNA end-processing, occurs throughout the cell cycle, and is the dominant repair pathway in the G0/G1 phase [18]. Briefly, the broken ends are bound by the Ku70/Ku80 dimer [19], which recruits DNA-dependent protein kinase catalytic subunit (DNA-PKcs), DNA Ligase IV, DNA polymerases, and associated scaffolding proteins to facilitate re-ligation of the broken ends [18].

By contrast, HR occurs in the presence of a homologous template, such as sister chromatids in the S/G2 phase, and utilizes DNA end resection and homology searching to resynthesize broken DNA and maintain the original sequence [20]. In HR, the Meiotic Recombination 11 (MRE11)-RAD50-Nibrin (NBS1) (MRN) complex initiates DNA end resection and processing of DNA ends in conjunction with CtBP-Interacting Protein (CtIP) [21,22]. After completion of this “short-range” end resection, the single-stranded DNA (ssDNA) generated is used as a platform for loading of exonuclease 1 (Exo1), the endonuclease DNA2, and Bloom Syndrome helicase (BLM), which coordinate further “long-range” resection to produce 3′ ssDNA overhangs [23]. Replication protein A (RPA) coats the ssDNA to stabilize it until mediator proteins like Breast Cancer Protein 1 (BRCA1), BRCA1-Associated RING Domain 1 (BARD1), Partner And Localizer of BRCA2 (PALB2), and Breast Cancer Protein 2 (BRCA2) facilitate RAD51 loading and formation of the RAD51 nucleoprotein filament [24,25,26,27], which mediates homology search and strand invasion to form a displacement loop (D-loop) [28,29]. This is followed by extension of the invading strand to restore its genetic sequence and gene conversion, which can proceed through multiple sub-pathways, including synthesis-dependent strand annealing and resolution of double Holliday junction intermediates [28,29]. For more in-depth discussion of the mechanisms of NHEJ, HR, and gene conversion sub-pathways, we refer readers to multiple thorough reviews [18,20,28,29].

#### 1.1.3. Chromosome Remodeling in DSB Repair

Repositioning of nucleosomes to allow access for repair was evidenced by early observations that nuclease sensitivity increases after UV-induced DNA damage in human fibroblasts [30]. This foundational work culminated in the “access–repair–restore” model [31], in which chromatin is first relaxed to allow access for DNA repair enzymes, and then restored after repair is completed. Much of this is accomplished by ATP-dependent chromatin remodeling complexes, which use the energy from ATP hydrolysis to catalyze the repositioning, removal, or exchange of histones in the nucleosome octamer [32]. In addition, enzymatic covalent modification of histone tails modulates histone dynamics directly, thereby altering accessibility [33]. These mechanisms often occur simultaneously and complement one another in remodeling chromatin surrounding a break.

However, a more nuanced understanding of chromatin has emerged in recent years, exploring the role of chromatin as a scaffold for signaling, recruitment, and modulation of the DNA damage response (DDR). This is exemplified by the discovery of damage-dependent phosphorylation of histone variant H2A.X [34] by kinases in the phosphoinositide-3-kinase-related protein kinase (PIKK) family [35], known as γH2AX. Phosphorylation of this histone variant starts a cascade of recruitment events that localize mediators of repair such as Mediator of DNA Damage Checkpoint 1 (MDC1) [36,37]. Since this discovery, chromatin biology has proven inextricably linked to regulated DNA repair in eukaryotes. Today, we view chromatin as both a barrier to enzymatic action and a key regulator of coordinated DNA repair. In this review, we discuss the major mechanisms of chromatin regulation in the context of DSB repair, summarize how these mechanisms are dysregulated in human disease, and highlight emergent concepts for future research.

## 2. Two Major Mechanisms of Chromatin Remodeling: Histone PTMs and ATP-Dependent Remodelers

### 2.1. Histone PTMs

**Figure 2:**
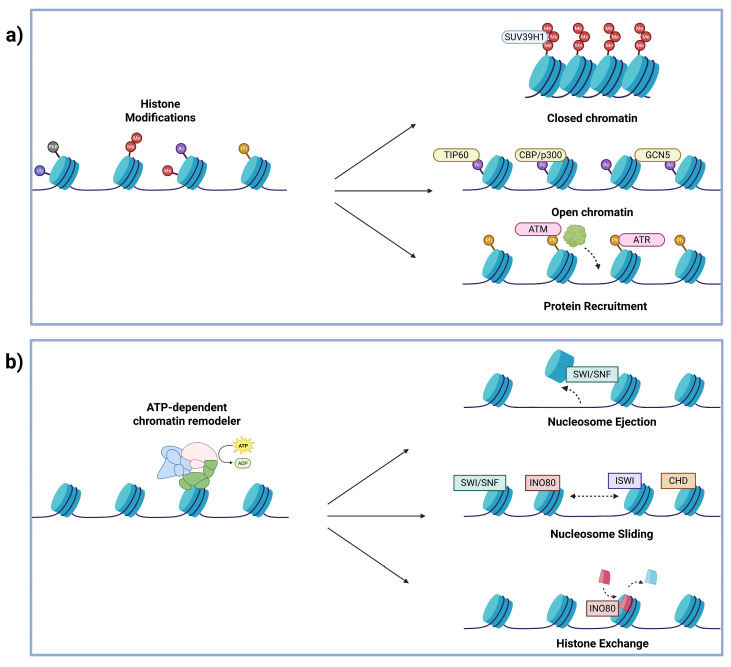
Chromatin remodeling through PTMs and ATP-dependent chromatin remodeling complexes. (**a**) Histone tails within the nucleosomes can undergo PTMs (Ex. ubiquitination [Ub], PARylation [PAR], methylation [Me], acetylation [Ac], and phosphorylation [Ph]). The type of modification can alter how the chromatin is remodeled. Enrichment of the repressive methylation mark H3K9me3 by SUV39H1 promotes chromatin compaction. Histone acetylation through TIP60 (H4K16 and H2K15), CBP/p300 (H4 (K5, K8, K12, and K16)), or GCN5 (H3K14) correlates with relaxed nucleosome spacing and open chromatin. ATM and ATR phosphorylate H2A.X at serine 139, recruiting DNA repair and chromatin remodeling proteins to damaged DNA. (**b**) ATP-dependent chromatin remodeling complexes use ATP hydrolysis to (I) eject nucleosomes (SWI/SNF family), (II) slide nucleosomes (SWI/SNF, INO80, ISWI, and CHD families), or (III) exchange histone variants (INO80 family) to remodel the chromatin, allowing for proper DNA-dependent pathways like replication, transcription, and DNA repair. Created in BioRender. Chiaramida, A. (2026) https://BioRender.com/e9eld1d.

One of the major mechanisms of chromatin remodeling in response to DSBs is through PTMs on histone tails that directly alter histone–DNA interactions and/or act as dynamic binding platforms for various proteins involved in repair. Many PTMs have been implicated in DSB signaling and repair, namely phosphorylation, PARylation, SUMOylation, ubiquitination, methylation, and acetylation (Figure 2a). Proteins that lay down these marks (writers), recognize them (readers), or remove them (erasers) play critical roles in regulating chromatin following DNA damage. In the following section, we will briefly review the canonical PTM mechanisms early in the DSB response and highlight key readers, writers, and erasers underlying this regulation.

#### 2.1.1. Phosphorylation and Ubiquitination Promote Canonical DSB Signaling

The MRN complex, consisting of MRE11, RAD50, and NBS1, is responsible for initial recognition of the DSB (via the DNA-binding activity of RAD50 [38]), which recruits and activates the key signaling kinase Ataxia-Telangiectasia Mutated (ATM) [39], and indirectly activates Ataxia-Telangiectasia and Rad3-related (ATR) [40]. These kinases phosphorylate many targets and are notable writers of serine 139 phosphorylation on histone variant H2A.X [34], which creates a binding platform for the reader MDC1 via its BRCA1 C-terminus (BRCT) domains [36,37]. MDC1 can then recruit additional MRN and ATM proteins to effectively “spread” the phosphorylation of H2A.X, known as γH2AX, as a signaling cascade for the recruitment of downstream effector proteins [36,41]. Additionally, MDC1 itself is phosphorylated by ATM, which promotes interaction with E3-ubiquitin ligase Ring Finger protein 8 (RNF8) via its Forkhead-Associated (FHA) domain [42]. RNF8 is a writer of ubiquitination in coordination with Ubiquitin Conjugating Enzyme E2 N (UBC13) [42] on H2A.X and potentially H2A [43]. Ring Finger protein 168 (RNF168), an additional ubiquitin ligase, binds ubiquitinated H2A and amplifies this signal [44]. This ubiquitin signal promotes the HR-mediator Breast Cancer gene 1 (BRCA1) to be recruited through the ubiquitin-binding motif on its interaction partner Receptor-associated protein 80 (RAP80) [42,43,44]. Additionally, the NHEJ-promoting Tumor suppressor p53-binding protein 1 (53BP1) is a reader of histone 2A lysine 15 ubiquitination (H2AK15Ub), catalyzed by RNF168, as well as histone 4 lysine 20 dimethylation (H4K20me2), present on bulk chromatin [45]. Owing to the importance of this ubiquitination event, RIDDLE syndrome is a genomic instability disorder characterized by an inactivating mutation in RNF168 that completely abolishes 53BP1 recruitment [46,47]. Additionally, the namesake of ATM, Ataxia Telangiectasia, is a multisystem disorder marked by neurodegeneration, immunodeficiency, and cancer predisposition caused by inactivating mutations in this key DDR kinase [48]. Thus, the signaling cascade initiated by ATM and amplified on an RNF8-RNF168 axis is essential for recruiting mediators of the two major DSB repair pathways, laying the groundwork for their well-established antagonism in repair pathway choice.

#### 2.1.2. Transient PARylation Promotes Early Recruitment and Chromatin Remodeling

While long recognized as key players in single-strand break (SSB) repair, it is now recognized that enzymes in the poly(ADP-ribose) polymerase (PARP) family are involved in DSB repair, where they catalyze poly(ADP-ribose) (PAR) addition to many substrates. PARP1, which is responsible for catalyzing 90% of PAR chains in response to DNA damage [49], is recruited to sites of DNA damage within seconds [50] via the DNA-binding activity of its zinc finger domains [51] and promotes MRN complex subunits MRE11 and NBS1 to be recruited [50]. Thus, it is debated whether PARP1 is a DSB sensor in addition to the canonical MRN and Ku70/Ku80 heterodimer sensors [51]. PARylation of the chromatin creates binding sites for many effector proteins for DSB repair and chromatin remodeling [51,52], and may directly promote chromatin relaxation through repulsion of the negatively charged PAR motif and DNA [51,53,54]. However, PAR residues are quickly degraded by the activity of de-ribosylase enzymes at the site of damage. Thus, PARylation represents a wave of transient signaling that acts early in DSB repair, whereas other modifications like γH2AX persist for continued signaling. PARylation modulates DSB signaling and chromatin remodeling, but loss of PARP delays but does not abolish ATM-dependent phosphorylation, highlighting some redundancy and interplay between these mechanisms [55].

#### 2.1.3. Methylation Regulates Pathway Choice

Histone methylation marks have been heavily implicated in DSB repair, both as regulators of pathway choice and of chromatin remodeling. Histone 4 lysine 20 mono- and di-methylation (H4K20me1/2), histone 3 lysine 36 di- and tri-methylation (H3K36me2/3), histone 3 lysine 9 trimethylation (H3K9me3), and histone 3 lysine 79 di- tri-methylation (H3K79me2/3) have been studied. In this section, we will highlight the unique role of H4K20me2 in the intersection between chromatin context and DSB pathway choice.

**Figure 3:**
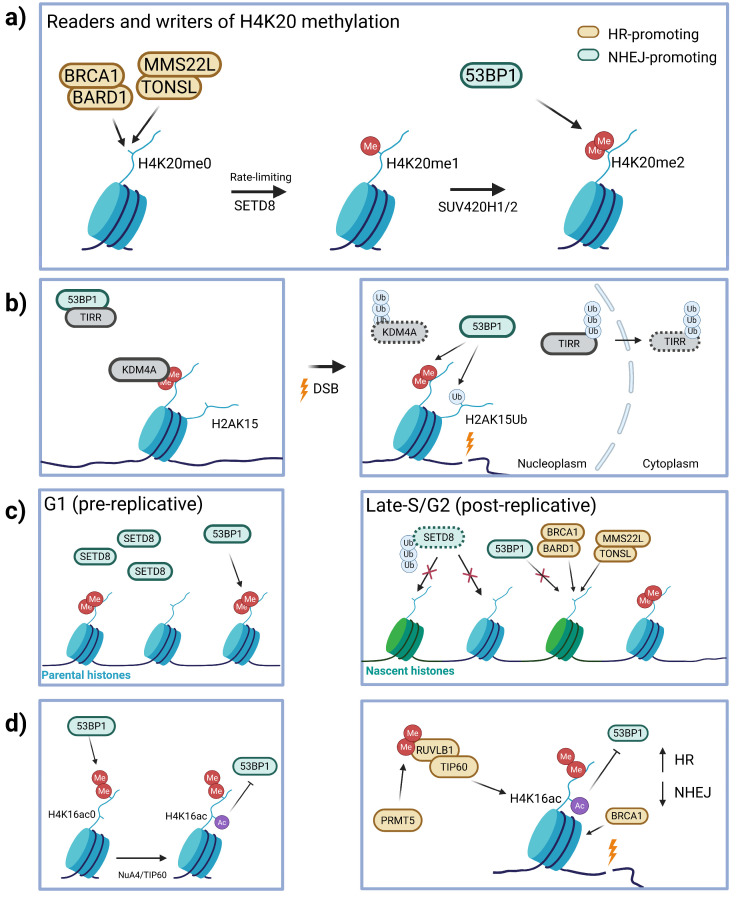
H4K20me2 as a regulatory hub for repair pathway choice. (**a**) The writers and readers of H4K20me marks. H4K20me0 is read by BARD1 and TONSL, both of which promote HR. H4K20me1 is catalyzed by SETD8, and H4K20me2 is catalyzed predominantly by SUV420H1/2. 53BP1 is a reader of H4K20me2. (**b**) Regulation of the accessibility of H4K20me2. At baseline, KDM4A binds to H4K20me2 and TIRR binds to 53BP1’s Tudor domains, both preventing an interaction between 53BP1 and H4K20me2. After damage, KDM4A is ubiquitinated and degraded, and TIRR is ubiquitinated and translocated to the cytoplasm for degradation. These events, coupled with RNF168-dependent H2AK15 ubiquitination, promote 53BP1 recruitment to damage sites. (**c**) Cell cycle regulation of H4K20me2. During G1, SETD8 levels are normal and H4K20me2 dominates. During replication, nascent histones are enriched in H4K20me0, which is maintained by ubiquitination and degradation of SETD8 in S phase. These events bias pathway choice toward HR on post-replicative chromatin. (**d**) NuA4/TIP60-dependent H4K16ac reduces 53BP1 binding affinity toward H4K20me2. 53BP1 reads and binds to H4K20me2. The acetyltransferase, NuA4/TIP60, acetylates H4K16, reducing the binding affinity of 53BP1’s tudor domain to H4K20me2. In the context of DSB repair, PRMT5-dependent methylation of TIP60’s cofactor RUVLB1 promotes TIP60-mediated H4K16ac and removal of 53BP1, thereby promoting HR. Created in BioRender. Chiaramida, A. (2026) https://BioRender.com/e9eld1d.

H4K20 methylation is central to the discussion of pathway choice in DSB repair, owing to 53BP1’s role as a reader of this mark through its tandem Tudor domains [45]. H4K20 mono- and di-methylation marks are written by Lysine Methyltransferase 5A (SETD8) [56,57] and Lysine Methyltransferase 5B/5C (SUV420H1/H2) [58], respectively, with monomethylation being a prerequisite for dimethylation (Figure 3a). Multiple mechanisms have been proposed by which H4K20me2 regulates repair pathway choice, primarily by modulating the accessibility and abundance of this dimethyl mark (Figure 3b,c). With regard to accessibility, Lysine-specific demethylase 4A (KDM4A) was identified as a competitive H4K20me2-binding factor that is degraded via RNF8-dependent ubiquitination following DNA damage [59]. It is thought that KDM4A binding limits H4K20me2 availability for 53BP1 binding, and its degradation after damage allows 53BP1 to bind. Conversely, Tudor-interacting repair regulator (TIRR) is well characterized to bind 53BP1’s Tudor domains and blocks its interaction with H4K20me2 [60]. It was recently found that TIRR is ubiquitinated and translocated to the cytoplasm for degradation following DSB induction, relieving the 53BP1-H4K20me2 block under damage conditions [61]. Together, these studies support a role for ubiquitination-dependent turnover of competitive binding factors in regulating the accessibility of the 53BP1–H4K20me2 interaction in response to damage. Notably, these H4K20me2-dependent mechanisms operate in parallel with RNF168-dependent ubiquitin signaling, underscoring that 53BP1 recruitment is controlled by combinatorial chromatin cues.

Abundance of the H4K20me2 mark is regulated by additional histone methyltransferases beyond SUV420H1/H2 that locally increase H4K20 methylation after DSB induction, such as dimethylation via Multiple Myeloma SET Domain (MMSET) [62] and monomethylation via SETD8. SETD8 is likely the rate-limiting factor in H4K20me2 deposition, being the sole enzyme responsible for monomethylation [57,63], and it has been shown to be required for proper 53BP1 loading [56,57]. Importantly, SETD8 is degraded during S phase via ubiquitination [64], but can be protected via the deubiquitinase Ubiqituin Specific Peptidase 29 (USP29) [65]. Similarly, quantitative image-based cytometry revealed that H4K20me2 is decreased in newly replicated chromatin, which directly correlates with reduced 53BP1 accumulation in the S phase [63]. Indeed, a recent study demonstrated that degradation-resistant SETD8 impairs HR in the S phase by maintaining high 53BP1 recruitment on newly replicated chromatin [66]. This agrees with evidence that H4K20me0 is read by the HR-promoting Tonsoku-like, DNA Repair Protein-MMS22L-like, DNA Repair Protein (TONSL-MMS22L) complex [67,68], and by the BRCA1 binding partner BARD1 [69] (Figure 3a). Taken together, this supports a model in which the lack of H4K20me2 is maintained partially by low levels of SETD8 in the S phase, antagonizing 53BP1 and recruiting BRCA1 via TONSL-MMS22L and BARD1 to bias newly replicated chromatin to be repaired by HR. Local induction of H4K20 monomethylation in response to IR can be mediated by USP29-dependent protection of SETD8 [65], raising the possibility that H4K20 methylation dynamics can be locally uncoupled from global cell-cycle regulation under damage conditions.

Owing to the importance of pathway choice along this axis, key players in this regulation have been investigated as therapeutic targets in human disease. SETD8 is overexpressed in multiple human cancers and is associated with poor outcomes, partially due to roles in transcriptional regulation and methylation of substrates like p53 and c-Myc [70,71,72]. Interestingly, IHC staining of cervical cancer tissue associated low SETD8 expression with cisplatin response in patients, and inhibiting SETD8 sensitized tumor xenografts to cisplatin [73]. Similarly, combining the alkylating agent melphalan and a SETD8 inhibitor exhibited synergistic effects in multiple myeloma cells by inducing DSBs [70]. Together, this suggests that SETD8 upregulation in cancer could promote therapeutic resistance to DNA-damaging agents by increasing NHEJ efficiency, and it will be interesting to further investigate whether inhibiting SETD8 can improve clinical outcomes in these cases.

#### 2.1.4. Acetylation Regulates DSB Repair

Histone acetylation marks are required for chromatin remodeling, contribute to DNA DSB repair, and are linked to DSB pathway choice. The marks are read by regulatory proteins containing a bromodomain (BRD) [74]. Histone acetyltransferases (HATs) acetylate lysines, thus neutralizing the residues’ positive charge. This reduces their interactions with negatively charged DNA and neighboring nucleosomes, producing a more open, dynamic chromatin structure that allows proteins and complexes to function during transcription, DNA repair, and replication [75,76,77]. Histone deacetylases (HDACs) remove this modification, compacting the chromatin. Here, we will discuss key acetylation marks, their respective modifiers, and their role during DSB repair.

#### 2.1.5. H4 Acetylation Through BRD-Containing Proteins

BRDs function as acetyl-lysine readers on chromatin and play important roles in DSB repair. Several BRD-containing proteins such as Bromodomain-containing protein 4 (BRD4), Zinc Finger MYND-type containing 8 (ZMYND8), ATP-utilizing chromatin assembly and remodeling factor (ACF1), Tripartite motif-containing 28/KRAB-associated protein-1 (TRIM28/KAP1), and Tripartite Motif-containing 33 (TRIM33) are recruited to DSB sites and participate in coordinating chromatin and repair activities [78,79]. Some HATs, including CREB-binding protein/p300 (CBP/p300) and General Control Non-repressed 5 (GCN5), also contain BRDs and localize to DNA lesions. CBP/p300 acetylates multiple lysines on H4 (K5, K8, K12, and K16) at DSB sites, which promotes recruitment of the NHEJ factors Ku70 and Ku80 and facilitates chromatin remodeling by enabling switch/sucrose non-fermentable (SWI/SNF) complex engagement [80]. By reading acetylation marks and recruiting remodelers and repair factors, BRD-containing proteins modulate histone acetylation, resulting in chromatin relaxation and targeted assembly of the repair machinery, thereby shaping repair pathway choice and efficiency.

#### 2.1.6. H4K16 and H2K15 Acetylation Through TIP60

Nucleosomal acetyltransferase of H4/Tat interactive protein-60 (NuA4/TIP60) is a multi-subunit HAT/chromatin remodeling complex that is highly conserved from yeast to human [77]. It acetylates core histones (H4 and H2A), histone variants (H2A.Z and H2A.X), and non-histone substrates such as p53 [81,82].

TIP60 modulates acetylation to promote HR. Mammalian cells lacking TIP60 or expressing catalytically inactive TIP60 are hypersensitive to ionizing radiation (IR) and accumulate unrepaired DSBs [83,84]. Depletion of TIP60 decreases BRCA1 foci formation, whereas 53BP1 increases at DSB sites [85]. TIP60-dependent acetylation of histone 4 lysine 16 (H4K16) reduces the binding affinity of the 53BP1 Tudor domain for H4K20me2 when present on the same H4 tail, thus determining the balance of BRCA1 and 53BP1 at DSB sites [85]. Later studies show in epithelial cells that Protein Arginine Methyltransferase 5 (PRMT5)-dependent methylation of the TIP60 coactivator, RuvB-like AAA ATPase 1 (RUVBL1), promotes TIP60-mediated acetylation of H4K16 and removal of 53BP1 from sites of damage (Figure 3d) [86]. Strikingly, in hematopoietic cells, loss of PRMT5 induces aberrant splicing of TIP60, which reduces global H4 acetylation and H2AK15ac. This loss in TIP60 acetylation impairs HR, increases 53BP1 foci, and sensitizes cells to PARP inhibitors [87]. TIP60 also acetylates H2AK15, which prevents the E3 ligase RNF168 from ubiquitinating the lysine, thus inhibiting 53BP1 binding and NHEJ during G2 and S phases [88]. These models suggest that acetylation through TIP60 is important for efficient HR and pathway choice.

#### 2.1.7. H3K56 Acetylation and Deacetylation During DSB Repair

The histone 3 lysine 56 acetylation (H3K56ac) modification regulates chromatin structure and function and is implicated in transcription, DNA replication, and repair [89]. H3K56ac increases in response to DSBs induced by IR and camptothecin (CPT), where the HAT CBP/p300 mediates H3K56 acetylation in response to damage in human cells [89,90]. However, conflicting studies implicate H3K56 deacetylation in DSB repair. The NAD+-dependent HDAC Sirtuin 6 (SIRT6), which has roles as a tumor suppressor, is linked to longevity, increases genomic stability, and is required for the recruitment of the chromatin remodeler Sucrose Nonfermenting 2 Homolog (SNF2H) to DNA DSBs [91,92,93,94]. By sliding the histone octamer, SNF2H mediates DNA accessibility [95]. SIRT6 promotes SNF2H binding by deacetylating H3K56 at damage sites, where loss of SIRT6 or mimicking persistent H3K56 acetylation (H3K56Q) prevents SNF2H-dependent chromatin opening and impairs downstream repair signaling, reducing recruitment of 53BP1, BRCA1, and RPA [94]. Efficient and accurate repair of DSBs is an emerging contributor to longevity [96]. Recently, bowhead whales, whose lifespan exceeds 200 years, were shown to repair DSBs more effectively than other mammals, suggesting that this improved genomic maintenance likely helps limit mutation accumulation and supports their exceptional lifespan [97]. The capacity for SIRT6 to promote DSB repair is also linked to longevity, where five amino acid changes in long-lived species account for increased efficiency of DSB repair [98]. Interestingly, a homozygous inactivating mutation in SIRT6 (D63H) results in severe congenital anomalies and perinatal lethality due to increased levels of H3K56ac and persistent stem cell transcription factor expression, thus dysregulating differentiation [99]. It would be interesting if pathogenic mutations in *SIRT6* are identified in the coming years, affecting its function during DSB repair.

Since H3K56ac levels both increase and decrease upon DNA damage, one study explored the role of the micro-environment on H3K56ac [100]. They showed that during DNA damage, both cell density and metabolite levels affect the dynamics of H3K56ac. In mammalian cells, H3K56ac levels increased as the cells grew from low density to high density [100]. They highlight how the NAD+-dependent HDACs Sirtuin 1 (SIRT1) and SIRT6 levels were higher in low cell density, where there was less H3K56ac compared to high cell density. These results were due to increased glycolytic activity and secretion of lactate as the culture became more confluent [100]. Extracellular metabolites seem to influence chromatin structure by modulating chromatin-modifying enzymes, which could explain the conflicting models that report H3K56 either being acetylated or deacetylated during DNA repair, but further work needs to address this.

### 2.2. ATP-Dependent Remodeling Complexes

**Figure 4:**
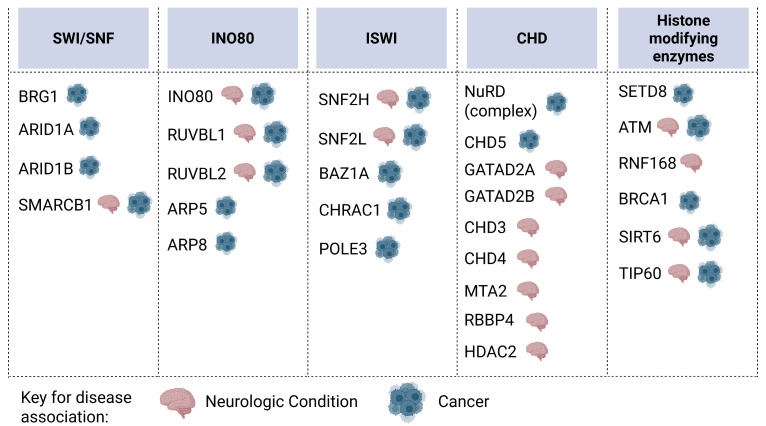
Disease associations of chromatin remodeling complexes and histone-modifying enzymes. Listed are representative subunits from the major chromatin remodeler families (SWI/SNF, INO80, ISWI, and CHD) and histone-modifying enzymes with their association to cancer (blue tumor) and/or neurological conditions (pink brain) as discussed in this review. Created in BioRender. Chiaramida, A. (2026) https://BioRender.com/e9eld1d.

Essential DNA-centric processes like replication, transcription, and repair depend on the accessibility of DNA to allow for protein complexes to initiate and carry out their processes [101,102,103]. ATP-dependent chromatin remodelers regulate DNA accessibility by using energy from ATP hydrolysis to position, eject, or exchange nucleosomes (Figure 2b). Eukaryotic cells contain four main families of chromatin remodelers, which are categorized based on their ATPase subunits, including SWI/SNF, INOsitol requiring 80 (INO80), imitation switch (ISWI), and chromodomain helicase DNA-binding (CHD) [102]. Dysregulation of these remodeling complexes is heavily dysregulated in cancer and neurodegenerative disorders (Figure 4). In this section, we will discuss the roles of the ATP-dependent remodeling complexes during DSB repair and the human diseases linked to their dysregulation.

#### 2.2.1. SWI/SNF Family

The yeast SWI/SNF and Remodeling the Structure of Chromatin (RSC) complexes were the first ATP-dependent chromatin remodelers to be characterized [104,105,106,107,108]. These complexes are evolutionarily conserved and correspond to the mammalian SWI/SNF (mSWI/SNF) family, which is assembled from products of 29 genes into three distinct complexes: canonical BAF (cBAF), polybromo-associated BAF (PBAF), and non-canonical BAF (ncBAF) [109,110]. Each complex shares some subunits while differing in others, and all include one of two ATPase catalytic subunits, Brahma-related gene 1 (BRG1 or SMARCA4) or Brahma (BRM or SMARCA2), which hydrolyze ATP to remodel chromatin via nucleosome sliding or eviction. The cBAF complex is characterized by the incorporation of one of the two AT-rich interactive domain-containing proteins (ARID), ARID1A or ARID1B, with the tandem plant homeodomain (PHD)-containing subunit Double PHD Fingers 2 (DPF2), which recognizes and interacts with acetylated histone tails. The PBAF complex contains ARID2, the BRD proteins Protein polybromo-1 (PBRM1) and Bromodomain-containing protein 7 (BRD7), and the PHD-containing PHD Finger Protein 10 (PHF10) subunit. The ncBAF complex is distinguished by its inclusion of the glioma tumor suppressor candidate region gene 1/GLTSCR1-like (GLTSCR1/GLSTCR1L) and BRD9 subunits [111].

##### BRG1

One of the most extensively studied subunits of mSWI/SNF in DNA damage repair is the ATPase catalytic subunit, BRG1. BRG1 recruits to sites of DNA DSBs and promotes repair by HR [112]. Knockdown of BRG1 in mammalian cells increases sensitivity to DSB-inducing agents such as etoposide and bleomycin and leads to the accumulation of unrepaired breaks. This is due to impaired RAD51 loading and retention of ssDNA and RPA at the break sites. The model proposed is that BRG1 facilitates HR by promoting the replacement of RPA with RAD51 via its mediator RAD52 [112].

Subsequent work confirmed and extended its role during HR mechanistically. The tumor suppressor retinoblastoma (RB) localizes to DSBs and recruits BRG1, promoting nucleosome remodeling that stimulates DNA end resection [113]. The same group later validated this model, showing that BRG1-deficient cells retain higher H3 occupancy and increased nucleosome density at DSBs, which impairs the recruitment of the nuclease, CtIP, and compromises DNA end resection [114].

BRG1 recruitment and activation at DSBs are coordinated with other chromatin regulators. PARP1 activity at breaks generates PAR chains that attract remodelers. One model described a PARP1–BRG1–SIRT1 axis in which PARP1-dependent PAR recruits BRG1 and SIRT1; SIRT1 deacetylates BRG1 to enhance its ATPase activity, and activated BRG1 clears nucleosomes to promote HR [115]. Together, these studies support a role for BRG1 at DSBs to stimulate end resection.

##### ARID1A and ARID1B

The ARID subunits of mSWI/SNF, ARID1A and ARID1B, are also implicated in DSB repair. ARID1A recruits to DNA breaks through an interaction between its C-terminal region and the ATR kinase [116]. Loss of ARID1A impairs HR and single-strand annealing, associated with reduced phosphorylation of RPA (Ser4/8) at DSB sites, consistent with a defect in DNA end resection [116]. Cells lacking ARID1A are also hypersensitive to PARP inhibition, further supporting a role in resection-dependent repair [116].

More recent work indicates that ARID1A contributes to both HR and NHEJ [117,118]. Bakr et al. proposed that ARID1A acts as an epigenetic regulator of DSB repair by promoting the formation of chromatin loops required for γH2AX focus formation. Using 4C-seq, they showed that both ARID1A and BRG1 are necessary to form a chromatin loop within the damaged topologically associating domain (TAD) [117]. Chromatin looping at DSBs is driven by cohesin-mediated loop extrusion, with the cohesin complex (SCC/RAD21) and the insulator protein CCCTC-binding factor (CTCF) flanking the break to permit ATM-dependent H2A.X phosphorylation [119,120]. ARID1A interacts with RAD21 and CTCF and facilitates their recruitment to DSBs, thereby promoting loop/TAD formation at damaged loci. They further report ARID1A represses local transcription by modulating activating histone marks (histone 3 lysine 27 acetylation [H3K27ac] and histone 2A lysine 118 acetylation [H2AK118ac]) and elongating RNA polymerase II (pS2-RNAPII) near transcription start sites at DSBs [117].

Complementing these findings, Kanno et al. show that ARID1A and ARID1B directly associate with DNA-PKcs via their armadillo (ARM) domains during late-stage NHEJ. Disruption of the DNA-PKcs binding residue (F3640A) impairs the ARID1A–DNA-PKcs interaction, compromises DSB repair, and reduces LIG4 recruitment [118].

ARID1B also contributes to DSB repair. ARID1B knockdown exacerbates etoposide-induced DNA damage, increasing levels of phosphorylated Checkpoint kinase 1 (pCHK1), pCHK2, pATM, and γH2AX, an effect attributed to increased chromatin accessibility upon ARID1B depletion [121]. Reduced ARID1B also activates the cGAS–STING pathway, linking ARID1B loss to innate immune signaling in response to DNA damage [121].

Together, these studies support important and multifaceted roles for ARID1A and ARID1B in coordinating chromatin architecture, DNA repair pathway choice, and the cellular response to DSBs.

##### SWI/SNF and Human Disease

Dysregulation of the SWI/SNF complex has been increasingly linked to human cancer. In BRG-mutated non-small-cell lung cancers (NSCLC), downregulation of the other ATPase, BRM, further sensitized cancer cells to IR [122], suggesting there is a synthetic lethal interaction between BRG and BRM, but more work will need to decipher whether this is cancer-specific. The highest mutation incidence in the SWI/SNF complex is in ARID1A, and up to 50% of ovarian clear cell carcinomas are mutated on this subunit [123]. Downregulation of ARID1A or ARID1B was shown to sensitize osteosarcoma cells and immortalized pancreatic ductal epithelial cells to DSB-inducing agents, such as IR and cisplatin [124,125].

Interestingly, SWI/SNF-related BAF chromatin remodeling complex subunit B1 (SMARCB1), also known as INI1, SNF5, or BAF47, is a core subunit of the SWI/SNF complex and acts as a tumor suppressor. Biallelic loss of SMARCB1 occurs in about 95% of malignant rhabdoid tumors (MRT), which primarily affect infants and young children and are accompanied by widespread changes in chromatin state [126]. *SMARCB1* lies at the distal end of chromosome 22q11.2, and deletions that include this gene are associated with the 22q11.2 distal deletion syndrome, a condition that is distinct from the classic 22q11.2 deletion, DiGeorge syndrome [127]. Patients with this distal deletion commonly present with prematurity, mild skeletal anomalies, growth delay, and developmental delay [128]. Because loss of SMARCB1 increases the risk of MRT, individuals with 22q11.2 distal deletions that include SMARCB1 are recommended to receive regular tumor surveillance [127,129]. Recently, a previously uncharacterized chromatin factor, also located on chromosome 22q11.2, zinc-finger protein 280A (*ZNF280A*), was shown to be essential for DSB repair. Mechanistically, ZNF280A binds to ssDNA via its zinc finger domains and enhances the recruitment of the BLM-DNA2 complex to DNA damage sites, facilitating long-range DNA end resection, a prerequisite for HR-mediated repair. Haploinsufficiency of the LCR22D-LCR22E region on 22q11.2, where the *ZNF280A* gene resides, directly links impaired DNA DSB repair to clinical features of 22q11.2 distal deletion syndrome [130]. Whether deletions of additional novel chromatin factors predispose patients to cancer is to be elucidated. Together, these findings underscore how loss of *SMARCB1* and haploinsufficiency of nearby chromatin factors such as *ZNF280A* on 22q11.2 may cooperatively disrupt chromatin regulation and DSB repair, thereby contributing to developmental defects and the marked predisposition to cancer in affected individuals.

#### 2.2.2. INO80 Chromatin Remodeling Complex

The mammalian INO80 complex is a multisubunit ATP-dependent chromatin remodeler built around the catalytic INO80 ATPase and a conserved core that coordinates nucleosome sliding, histone variant exchange, and interactions with regulatory factors. Core components include the INO80 ATPase itself and the AAA+ ATPases TIP49a/b (RUVBL1/RUVBL2), which contribute to complex assembly and stability [131]. Actin-related proteins ARP4, ARP5, and ARP8 facilitate chromatin engagement and remodeling activity [132,133]. Mammalian-specific accessory subunits such as Nuclear Factor Related to KappaB Binding Protein (NFRKB) and other INO80-associated factors mediate genomic targeting and coupling to transcriptional regulators [134,135,136]. The combinatorial subunit architecture and regulatory PTMs thus tune INO80’s ATP-driven remodeling activity to support diverse processes in genome maintenance and gene regulation.

The mammalian INO80 complex was initially reported to be recruited to laser-induced DSBs in a γH2AX-independent manner, a distinction from yeast [137,138]. Subsequent work discovered that INO80 recruitment to DSBs is required for efficient HR, where knockdown of INO80 produced defects in DDR, with reductions in 53BP1, RPA, and BrdU foci following IR, implicating INO80 in DNA end resection [139]. Notably, RAD51 recruitment was not decreased in these experiments, suggesting that INO80 may not be essential for downstream HR events [139].

Later studies produced contrasting results about the stage at which INO80 functions. One report shows that INO80 depletion reduces RAD51 foci, but does not affect RPA foci after IR, implying a role for INO80 beyond resection [140]. Differences between studies may reflect cell-line-specific effects, experimental conditions, or methodologies. They further show that INO80 removes the histone variant H2A.Z from chromatin flanking DSBs. H2A.Z is rapidly incorporated at damage sites catalyzed by the p400 motor ATPase subunit of NuA4, and removal promotes HR [140]. Histone exchange rapidly remodels chromatin at sites of damage, increasing local accessibility so that sensors and repair factors can engage with the chromatin. H2A.Z exchange is required for proper loading of Ku70/80 and restricts resection, highlighting the importance of its regulation for pathway choice [141]. Using sister chromatid exchange (SCE) as a readout of HR completion, they observed fewer SCEs in INO80-deficient cells, consistent with a contribution of INO80 to downstream HR steps following resection [140].

Recent work highlights a role for the INO80 chromatin-remodeling complex in coordinating DSB repair with neural progenitor cell (NPC) behavior during corticogenesis [142]. They used conditional Ino80 deletion in mice cortical NPCs to show that loss of INO80 leads to accumulation of unrepaired DSBs, p53 activation, apoptosis of progenitors, and microcephaly [142]. Mechanistically, INO80 deficiency produced a marked reduction in HR relative to NHEJ as measured by an in vivo CRISPR–Cas9 repair-pathway choice assay, while transcriptomic analyses showed little change in the expression of core HR genes. Co-deletion of Transformation-related protein 53 (*Trp53*) rescued the apoptotic and anatomical phenotypes, indicating that the major developmental consequences of INO80 loss stem from impaired HR and subsequent p53-dependent responses rather than from broad transcriptional dysregulation [142]. These findings position INO80 as a chromatin-based regulator of repair-pathway utilization in stem cells and implicate chromatin dysregulation of HR as a potential mechanism contributing to developmental brain disorders.

Evidence for a specific role of mammalian INO80 in NHEJ is limited. One study suggests that INO80 promotes nucleosome disassembly independently from DNA end resection, where INO80 depletion led to faster accumulation of DNA breaks and a delayed decrease in H3 occupancy around DSBs [143]. However, additional work is needed to define any direct function of mammalian INO80 in NHEJ.

##### ARP8

Multiple studies implicate the ARP8 subunit in DSB repair and INO80 function. Knockdown of ARP8, but not other ARP subunits, impaired recruitment of the INO80 complex to laser-induced DNA damage [137]. ARP8 is linked to DNA end resection, where its depletion reduces RPA foci after IR [139]. ARP4 and ARP8 directly bind core histones and contribute to chromatin remodeling [132,144]. Building on this, other studies show that ARP8 binds double-stranded DNA (dsDNA) and preferentially ssDNA, and ARP8 knockout increases γH2AX foci after treatment with the DSB-inducing agent CPT. They proposed that ARP8 supports INO80-mediated chromatin remodeling to enable proper DNA end resection [133].

Recent yeast studies identified the Casein kinase 1 (CK1) family kinases, casein kinase I homologs (Hhp1 and Hhp2), as ARP8 kinases required for proper DSB repair, where loss of Hhp1/Hhp2 phosphorylation compromised ARP8 function during DNA damage [145]. Although phosphorylation of ARP8 was not demonstrated in a mammalian model, inhibition of CK1δ/CK1ε in HeLa cells prolonged DSB persistence, consistent with a conserved role for CK1 activity in repair [145]. Given the evolutionary conservation of DNA repair pathways and CK1 functions, it will be important to test whether ARP8 phosphorylation by CK1 homologs likewise regulates INO80 and DSB repair in mammals.

##### INO80 and Human Disease

INO80 complex dysfunction is implicated in human disease, particularly cancer and disorders of genome stability. Altered expression or mutation of *INO80* subunits is reported across multiple tumor types and correlates with genomic instability, altered DDR, and treatment sensitivity [116,135]. Decreased INO80 levels promote chromosome segregation defects and DNA damage accumulation, while INO80 overexpression is linked to tumor proliferation and poor prognosis in some contexts [146,147]. Loss or mutation of *ARP8* and other INO80 components sensitizes cells to genotoxic agents and to PARP inhibition, suggesting potential therapeutic vulnerabilities [138,139]. Beyond cancer, perturbation of INO80-regulated chromatin remodeling is connected to neurodevelopmental phenotypes and chromosomal instability in model systems, supporting a broader role for INO80 in maintaining genome integrity in humans [142,148]. Together, these studies indicate that INO80 status can influence disease etiology and response to DNA-damage therapies, thus motivating further characterization of INO80 alterations in patient cohorts.

Dysregulation of INO80 is increasingly linked to human cancer. Elevated INO80 expression is reported in NSCLC and colon cancer. In NSCLC, INO80 is highly expressed and correlates with enhanced proliferation, tumor formation, and increased genome accessibility, leading to enhanced expression of downstream genes [147]. In colon cancer, INO80 demonstrates a high rate of amplification and expression [146]; however, the mechanisms of INO80 function are elusive. A threat to genome stability is conflicts between the DNA replication and transcription machinery, leading to DSBs [149]. Co-transcriptional RNA:DNA hybrid structures known as R-loops are an obstacle to replication fork progression. In cancer cells, R-loops are highly abundant, yet they sustain sufficient DNA synthesis to proliferate. INO80 was found to be recruited to R-loop-enriched sites and increased turnover to promote proper replication and increased proliferation in cancer cells [150]. A haploinsufficient mutation of *INO80* inhibits colon cancer tumorigenesis by activating ATR-Chk1 signaling, inducing stalled replication forks, and increasing apoptosis [146]. Collectively, these studies suggest that INO80 represents a potential therapeutic vulnerability in cancers that rely on elevated chromatin remodeling and DNA damage tolerance for continued growth.

#### 2.2.3. ISWI Family Remodeling Complexes

ISWI family remodeling complexes are conserved across species [151]. There are 7 different mammalian ISWI complexes discovered so far, which are Williams Syndrome Transcription Factor-ISWI (WICH), Nucleolar remodeling complex (NoRC), Remodeling and Spacing Factor (RSF), ATP-utilizing chromatin assembly and remodeling factor (ACF), Chromatin Accessibility Complex (CHRAC), Nucleosome Remodeling Factor (NURF), and Chromatin Remodeling containing factor (CERF). Each of these complexes contains the ATPase SNF2H, while NURF and CERF contain SNF2L [151]. ISWI plays a role in SSB repair and DSB repair. Here, we will talk about the role of ISWI remodeling complexes during DSB repair and the human diseases associated with these complexes.

##### SNF2H

The ATPase SNF2H is implicated in DSB repair. SNF2H accumulates to DSBs, and its depletion leads to sensitivity to DSB-inducing agents in human cells and impairs both HR and NHEJ [152]. H2B monoubiquitination by RNF20 is followed by H3K4 methylation at DSB sites, which in turn recruits SNF2H to DSB sites. Depletion of SNF2H led to disrupted RPA, RAD51, and BRCA1 foci after IR induction [153].

Later studies build on SNF2H’s role in DSB repair. Following DNA damage, the NAD+-dependent HDAC SIRT6 recruits to DSB sites to deacetylate H3K56 and specifically recruits SNF2H to open condensed chromatin for the proper recruitment of both NHEJ and HR DDR proteins [94]. SNF2H and SIRT6 also work together to stabilize H2A.X during DSB repair [154]. H2A.X is rapidly turned over under normal conditions, but in response to DSBs, H2A.X is transiently stabilized and incorporated into chromatin. ATM and SIRT6/SNF2H work together to rapidly stabilize H2A.X upon DSB formation by blocking the E3 ligase HUWE1 from ubiquitinating and degrading H2A.X.

Another group observed that SNF2H recruits to DSB through PARP1. When PARP was inhibited, there was a reduction in the spreading of SNF2H to laser-induced damage sites [155]. They also showed that BRCA1 recruitment was impaired in the presence of catalytically dead SNF2H [155]. Another group reported that the structural nuclear mitotic apparatus protein (NuMA) accumulates at sites of DNA damage in a PARP-dependent manner and interacts with SNF2H, regulating its diffusion in the nucleus and its accumulation at DSB sites [156].

##### ACF1 and RSF1

The ACF1 complex also plays a role during DSB repair. ACF1 recruits to DSB sites, where it interacts with Ku70, allowing for the accumulation of Ku proteins at DSB sites to enhance NHEJ [152]. The RSF complex is made up of RSF1 and the SNF2H ATPase [151] and is implicated in DSB repair. RSF1 regulates the ATM-dependent signaling pathway and DNA repair through HR and NHEJ [157]. Later studies show that RSF1 recruits HDAC1 to deacetylate H2A.X-K118 at DSB sites, enabling ubiquitination of H2A-K119. This ubiquitination silences transcription at DSBs, permits γH2AX propagation, and promotes DSB repair [158].

##### ISWI and Human Disease

Dysregulation of ISWI chromatin remodeling complexes is implicated in multiple cancer types. The ATPase subunit SNF2H is overexpressed in breast and ovarian cancers, as well as in acute myeloid leukemia (AML), where elevated expression levels correlate with increased tumor cell proliferation, larger tumor size, and poorer overall survival [159]. Other subunits like Bromodomain Adjacent to Zinc Finger Domain 1A (BAZ1A), CHRAC1, and DNA Polymerase Epsilon 3 (POLE3) are also upregulated in a variety of tumors [159]. However, despite substantial evidence linking ISWI complex amplification or overexpression to oncogenesis, most studies remain largely correlative and do not fully elucidate the molecular mechanisms by which these remodelers drive cancer progression.

#### 2.2.4. CHD Remodeling Complexes

The CHD family of chromatin remodeling complexes is defined by the presence of dual N-terminal chromodomains in the core ATPase, which are epigenetic readers of methylated lysine residues on histone tails [160]. There are nine ATPases in this family (CHD1-9), categorized into three subfamilies [161], some of which function as monomers, while others act in complex with additional subunits. Subfamily I (CHD1 and 2) members contain DNA-binding domains, whereas subfamily II (CHD3, 4, and 5) do not, instead harboring PHD zinc fingers N-terminal to the chromodomains. Subfamily III (CHD6-9) is the least studied, marked by two C-terminal Brahma and Kismet (BRK) domains [161,162]. In the following section, the role of CHD3, CHD4, and CHD5 will be highlighted.

##### NuRD Complexes

The best studied role of CHD family members in DNA repair is through the Nucleosome Remodeling and Deacetylase (NuRD) complexes, which contain CHD3, CHD4, or CHD5 and possess HDAC activity through HDAC1 and/or HDAC2 subunits within the complex. For instance, CHD3 is bound to the heterochromatin adaptor KAP1 through interactions with a conjugated SUMO1 on KAP1, promoting tight chromosomal compaction. Induction of a DSB causes ATM-dependent phosphorylation of KAP1, which disrupts the interaction between CHD3 and KAP1, resulting in the removal of CHD3 near the break site and subsequent chromatin relaxation for DSB repair [163,164,165,166]. This suggests that CHD3 supports heterochromatin formation under homeostasis, but its removal following DSB induction enables chromatin relaxation necessary for repair initiation. By contrast, CHD4 is recruited in a PARP-dependent manner to DSBs to promote histone ubiquitination within the RNF8/RNF168 axis [167,168,169,170], and can interact with BRCT-repeat Inhibitor of hTERT Expression (BRIT1) to regulate BRCA1 loading and facilitate [171]. In addition, the HDAC SIRT6 regulates recruitment of the CHD4 remodeler to compacted DSB regions in G2 [172]. SIRT6 and CHD4 competitively bind to H3K9me3 to displace heterochromatin protein 1 (HP1), promote local chromatin decompaction, and thereby facilitate HR [172]. Thus, CHD3- and CHD4-containing NuRD complexes possess distinct, and in some settings, opposing mechanisms of nucleosomal reorganization to regulate DSB repair.

CHD5 is a known tumor suppressor frequently deleted in neuroblastoma that is predominantly expressed in brain tissues [173,174,175]. It has been shown to associate with NuRD complex subunits, indicating a potential overlap with other CHD family members, but its potential role in DNA repair has been largely unexplored. To date, only one study has reported that siRNA knockdown of CHD5 in pancreatic cancer cells increases baseline γH2AX and phosphorylated Chk2, suggesting impaired genomic maintenance [176]. Further work will be necessary to elucidate whether CHD5-containing NuRD complexes directly promote DSB repair, either redundantly with CHD3 or CHD4, or by a unique mechanism.

##### GATAD2A and GATAD2B

Beyond the core ATPase, the inclusion or exclusion of additional subunits in the NuRD complex has been explored, namely the mutually exclusive GATA Zinc Finger Domain Containing 2A (GATAD2A)-containing and GATA Zinc Finger Domain Containing 2B (GATAD2B)-containing complexes. One study found that GATAD2A NuRD complexes participate in substoichiometric interactions with ZMYND8, which coordinate the complex to zinc finger proteins that facilitate binding to DSB sites to direct repair [177]. By contrast, a recent study linked GATAD2B-containing NuRD complexes to chromatin compaction by binding DNA:RNA hybrids generated peripheral to DSBs and promoting histone deacetylation [178]. This peripheral compaction is thought to create a barrier within which chromatin relaxation can be locally achieved for DSB repair without excessive hyperrelaxation. Therefore, various NuRD complexes recruited by distinct mechanisms may fine-tune the local chromatin environment for optimal DSB repair.

##### CHD and Human Disease

Dysfunction of NuRD complexes is present in multiple human pathologies. Deleterious mutations in NuRD subunits such as *GATAD2A/B* and *CHD3/4* have been identified in cases of neurodevelopmental disorders characterized by reduced communicative function, intellectual disability, facial dysmorphisms, macrocephaly, and global developmental delays [179,180]. Additionally, the NuRD subunit *GATAD2A* has been associated via genome-wide association studies (GWAS) with increased schizophrenia risk, and additional subunits (*CHD4*, Metastasis-Associated 1 Family Member 2 [*MTA2*], RB binding protein 4 [*RBBP4*], and *HDAC2*) have been associated with bipolar disorder [179].

This dysfunction has been explored in the context of NuRD as an epigenetic regulator in the fetal brain, governing cell fate decisions in brain development [179]. While there is very strong evidence supporting this model, it will be interesting to uncouple this classical function from the emergent role of NuRD in regulating DSB repair. Indeed, clinical presentations of global developmental delay, intellectual disability, and facial dysmorphisms are key features of disorders underpinned by genomic instability, as accumulating DNA damage promotes cellular dysfunction. The complicated relationship between chromatin remodeling to regulate transcription and as a DNA repair mechanism is exemplified by NuRD’s contradictory role in cancer, acting both to promote tumorigenesis and suppress cancer progression, depending on the context [181]. Clearly, one function cannot be explored without the other, and the specific NuRD subunit composition, tissue expression, developmental stage, and disease pathology must be considered when investigating these disorders.

## 3. Emerging Concepts and Unanswered Questions

### 3.1. Higher Order Structure

Beyond the local action of nucleosome remodeling and histone modifications, larger-scale genomic rearrangements in 3-dimensional space have been investigated as regulators of processes like transcription, replication, and DNA repair [182]. Briefly, chromatin folding results in the formation of two major functional compartments in interphase that physically associate in the nucleus: the euchromatin (A) compartment characterized by active transcription, and the condensed heterochromatin (B) compartment. Within these compartments, the action of architectural proteins and barrier elements facilitates the formation of self-interacting topologically associating domains (TADs) that coordinate the function of genomic regions by 3D proximity. In the following sections, we will review the emergent role of higher-order architecture in DSB repair, namely (1) DSB clustering and chromatin compartmentalization and (2) differences between heterochromatic and euchromatic repair. Finally, we will present our current understanding of chromatin reestablishment following DSB repair.

#### 3.1.1. DSB Clustering

Evidence that damaged chromatin domains exhibit mobility and form clusters when DSBs are induced in human cells was first reported in 2004 [183] and further explored in 2017 [184] by using Hi-C, finding that this is a property of breaks in transcribed genes in the A compartment [184,185]. DSB clustering is observed predominantly in the G1 phase and results in delayed repair, namely, in breaks that would typically undergo HR in G2 [184,186,187]. Mechanistically, clustering has been linked to actin polymerization, the MRN complex, and the linker of nucleoskeleton and cytoskeleton (LINC) complex [186,187]. DSB clustering may serve multiple functions, one of which could be to delay repair of complex breaks in transcribed regions during G1, when HR is unavailable, influencing pathway choice for later HR repair in a post-replicative state. Future studies of the interplay between pathway choice and cluster formation are necessary.

#### 3.1.2. TADs, Cohesin, and Compartments

**Figure 5:**
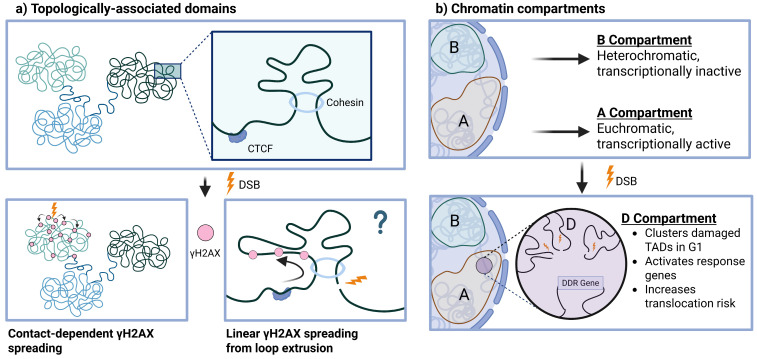
Emerging concepts linking higher-order genomic architecture to DNA double-strand break repair. (**a**) Chromatin is organized into self-interacting topologically associated domains (TADs) that coordinate genomic function. These are formed via cohesin-mediated loop extrusion, a process regulated by CTCF binding to boundary elements and creating a barrier to loop extrusion. In DSB repair, there is strong evidence that the γH2AX signal corresponds to spatial proximity, creating a damaged TAD that recruits repair factors. Additionally, there is evidence that loop extrusion may be induced in response to DSBs, resulting in linear γH2AX spreading as nucleosomes pass through the loop. The degree to which this mechanism is relevant and contributes to γH2AX spreading is still unclear, as denoted by the "?" symbol. (**b**) Chromatin compartments are large spatial segregations of either active (A compartment) or inactive (B compartment) domains that self-interact. There is evidence that in response to a DSB, a subcompartment within the A compartment arises, referred to as the D compartment, which is thought to regulate the DDR. The D compartment clusters DSBs in spatial proximity mainly in G1 and segregates a subset of DDR genes (correlating with their activation), but poses an increased risk of translocation due to the spatial proximity of breaks. Created in BioRender. Chiaramida, A. (2026) https://BioRender.com/e9eld1d.

Further work has implicated TADs in the DDR, namely, in the generation of γH2AX signaling hubs by two complementary mechanisms: (1) contact-dependent [188] and (2) loop extrusion-mediated spreading (Figure 5a) [119]. In the contact-dependent model, the physical proximity of nearby chromatin to the DSB, orchestrated by the chromatin architectural protein CTCF, determines the resultant γH2AX spreading, typically but not strictly, within TAD boundaries. This model of spreading is not linear across the chromatin and is supported by evidence that γH2AX spreading correlates with pre-existing TAD structure. In the loop extrusion model, cohesin is recruited near DSBs and mediates loop extrusion within a damaged TAD, and ATM phosphorylates H2A.X as the chromatin is looped through linearly.

Collectively, the mechanisms of DSB clustering and induction of signaling within damaged TADs have led to the definition of a DSB-induced higher-order chromatin compartment, referred to as the D compartment that self-segregates after damage and correlates with upregulation of a subset of DDR genes, thus modulating the response to persistent DSBs (Figure 5b) [185]. While this clustering and higher-order rearrangement may facilitate DSB repair, it also increases erroneous translocation events from the proximity of broken chromosomes [185,187], explaining some translocations observed in cancer genomes [185]. Thus, it will be interesting to consider if DSB clustering and chromatin folding are adaptive or maladaptive in certain contexts, and how they might be dysregulated in disease.

#### 3.1.3. Two Waves of DSB Clustering

Liquid–liquid phase separation (LLPS) has been implicated as a potential mechanism of early DDR organization. Briefly, LLPS is the demixing of a liquid solution into multiple distinct phases, driven by weak, multivalent interactions that overcome the entropy of mixing in solution [189]. It is characterized by spherical morphology, fusion dynamics, rapid exchange within the condensate, selective permeability between the condensate and the surrounding liquid, and concentration dependence. The clustering dynamics of DSBs have prompted discussion of whether DSB clustering is mediated in part by a phase separation mechanism. Indeed, early accumulations of 53BP1 exhibit hallmarks of LLPS: droplet-like fusion and fission, phase-permeability barrier via half-FRAP, and 1,6-hexanediol sensitivity [185,190,191,192].

However, the physiologic relevance of LLPS is highly debated [193], and late-clustered 53BP1 foci lack the phase barrier and spherical shape essential for LLPS [185]. Recent kinetic analysis using laser microirradiation suggests that DSB clusters occur in two temporally distinct phases, with early clustering (0–5 min) correlated with faster repair and secondary clustering (20–60 min) with delayed repair [194]. Rather than representing mutually exclusive mechanisms, phase-separation and structural compartmentalization may represent two temporal stages of DSB clustering. These observations suggest a model in which local, early clustering is mediated by LLPS to form an immediate condensate that initiates the DDR, but persistent breaks in G1 are clustered at later timepoints via architectural reorganization to facilitate accurate repair by HR. Further work will be needed to determine if this transition represents a true biological phenotype or an artifact of the experimental systems used (laser- or endonuclease-induced breaks, cell line variation, and microscopy resolution limits).

#### 3.1.4. Recent Contradictions of DSB-Induced Loop Extrusion

Loop extrusion as a mechanism of γH2AX spreading, while enticing, is contested by conflicting reports about the function of cohesin in recent years. There is agreement that cohesin is enriched near DSB sites (at pre-existing CTCF motifs) [195,196], phosphorylated by ATM [196,197], and that its depletion delays DSB repair [195,197], but there is significant contradiction in its importance for γH2AX spreading and disagreement as to whether loop extrusion is increased in response to DSBs [119,195,198,199].

Still, recent work using degron-mediated depletion of cohesin suggests that the basal activity of cohesin in organizing chromatin into TADs protects against NHEJ *cis* translocations [195]. Additionally, the role of cohesin phosphorylation is not well understood mechanistically, but multiple studies suggest that cohesin likely plays roles which are dependent and independent of its phosphorylation [195,197]. It has proven difficult to tease apart the fundamental baseline architectural roles of cohesin from damage-induced roles. As aptly noted [195,200], there are major caveats in studying cohesin in DNA repair. First, the mechanism of cohesin depletion (siRNA or degron) may bias results owing to the high stability of cohesin subunit proteins in the nucleus. Second, the role of cohesin as a known architectural protein and a phosphorylation target of ATM has not been extensively clarified. Third, cohesin exists in “extrusive” and “cohesive” complexes that perform different architectural functions. Both forms of cohesin have been recently implicated in the RAD51-dependent homology search [201,202], further complicating attempts to deplete cohesin and determine its functional impact. Rigorous work is required to tease out these complexities and develop a consistent model of cohesin’s role in DSB repair that takes these factors into account.

Lastly, one of the biggest limitations across these and most other DNA repair studies is the use of a few human cell lines for the vast majority of experiments, namely, the osteosarcoma U2OS cell line, the cervical cancer HeLa cell line, and the immortalized retinal pigment epithelial hTERT-RPE1 cell line. U2OS contains a large deletion in the chromatin remodeler *ATRX*, which allows it to utilize a homology-directed repair pathway known as Alternative Lengthening of Telomeres (ALT), present in some cancers that do not reactivate telomerase [203,204]. HPV viral protein E6 mediates degradation of p53 in HeLa cells, meaning cell cycle checkpoints and apoptosis pathways are often defective in this cell line [205]. Notably, both cell lines also have an abnormal karyotype. By contrast, non-cancerous RPE-1 cells are diploid and more genomically stable compared to cancer cell lines [206]. Use of these cell lines in combination strengthens the generalizability of DNA repair discoveries, but it is important to consider tissue-specific contexts as well. For instance, while the *BRCA1* mutation confers a high risk of certain cancers (breast and ovarian) in germline carriers, it poses only a minor risk in other tissues [207]. It is possible that chromatin organization within different tissue types necessitates differential mechanisms of DSB repair regulation. Thus, studying DNA damage within a disease context yields the best chance of generating clinically relevant findings for that disease.

### 3.2. Repair in Heterochromatin

Within the access–repair–restore model, compact heterochromatin is viewed as a challenge to DSB repair, limiting accessibility of required enzymes. Heterochromatic regions include constitutive heterochromatin, which is enriched at centromeres marked by Centromere Protein A (CENP-A) and pericentromeres marked by H3K9me3 and HP1, as well as facultative heterochromatin, which is marked by histone 3 lysine 27 trimethylation (H3K27me3) [208]. In addition, a lack of histone acetylation marks also denotes compacted heterochromatic regions. Although our understanding of DSB repair in heterochromatic regions is incomplete, it has been a major subject of study in recent years.

Understanding complexities between heterochromatic and euchromatic repair has been difficult since global damage induction can obscure subtle differences between genomic regions, and certain types of DSB induction such as endonucleases bias toward creating breaks in accessible chromatin. Most studies of heterochromatic repair have been conducted in mouse or *Drosophila*, owing to their DAPI-rich, heterochromatic chromocenters easily visualized under immunofluorescence microscopy. Still, it is well understood that repair of DSBs arising in euchromatin and heterochromatin exhibits distinct repair characteristics.

#### 3.2.1. Constitutive Heterochromatin

One of the major themes of DSB repair in constitutive heterochromatin is break mobility. In organisms with prominent chromocenters, including *Drosophila* and mouse, DSB relocalization has been well documented, supporting a model in which clustered repeats pose a high risk for aberrant recombination and therefore necessitate spatial repositioning of lesions to safer nuclear compartments. In these systems, breaks are relocated to the periphery of pericentromeric domains (mouse) or to the nuclear periphery (*Drosophila*). For a thorough review of these mechanisms across species, we refer readers to [209].

In contrast, heterochromatic DSBs in human cells appear substantially less mobile, as recently studied in pericentromeric breaks [210], which were positionally stable and able to recruit HR factors. This is consistent with the absence of large chromocenter structures and reduced interchromosomal clustering of repetitive sequences. Instead, heterochromatic breaks in human cells are locally relaxed in an ATM-dependent manner. Briefly, ATM phosphorylates KAP1, a key corepressor enriched at H3K9me3 sites, which causes release of the nucleosome remodeler CHD3, resulting in chromatin decompaction [163,164,165,166]. The defect in DSB repair observed by ATM inhibition can be reversed by knockdown of KAP1, HP1, or HDAC1/2 [163], providing strong evidence that chromatin relaxation is a key step in repair of heterochromatic breaks.

The barrier to repair imposed by constitutive heterochromatin is exemplified by sequencing of cancer genomes, which shows higher mutational rates in heterochromatic regions, strongly correlating with H3K9me3 across multiple studies [211,212]. This is consistent with less efficient repair and error-prone processing of DSBs. More work needs to be done to establish additional regulators of repair in human heterochromatin and to delineate between repair in pericentromeres and centromeres. For instance, recent studies of DSBs in human centromeres show that HR can be activated in G1 in these structures [213], with the suggestion that centromeric DNA breaks and subsequent repair by HR may function to maintain the epigenetic profile of centromeres, reinforcing CENP-A occupancy at these sites [214] owing to CENP-A’s documented recruitment to DSBs [215].

#### 3.2.2. Facultative Heterochromatin

Facultative heterochromatin, which is characterized by H3K27me3 and the repressive polycomb complex, serves important functions in gene regulation and is significantly more dynamic compared to constitutive heterochromatin. Although the transient deposition of H3K27me3 at breaks in euchromatin [167] is one of multiple mechanisms known to induce transcriptional silencing at DSBs [216], research on DSBs originating in facultative heterochromatin is lacking. The repair of DSBs in facultative heterochromatin has mainly been investigated using the inactive X chromosome as a model, finding DSB-induced decondensation around the inactive X chromosome in female fibroblast cells with some suggestion of break mobility [217]. Similarly, a recent study in *Drosophila* has indicated that breaks within facultative heterochromatin relocalize outside of polycomb bodies to facilitate repair, but this should be cautiously extrapolated to human cells due to previously mentioned differences in heterochromatic structure across species [218]. For more discussion of DNA repair in facultative heterochromatin, we refer readers to [219].

#### 3.2.3. Positive Roles of Heterochromatin Proteins in DSB Repair

Seemingly conflicting results have been reported about key players in constitutive heterochromatin, such as KAP1, HP1, and H3K9me3. One source of confusion is the fundamental difference between *Drosophila* and mouse chromocenters, which cluster interchromosomal heterochromatin, and human heterochromatin, which does form chromocenters. Studies of HP1 isoforms in other species [210,220,221,222,223] suggest that HP1-rich chromocenter domains expand and restrict access of HR machinery until breaks are relocated to permissive nuclear compartments, as previously discussed. This should be interpreted cautiously with respect to human heterochromatin, which does not exhibit the same mobility kinetics of DSBs in heterochromatin as in other species [210]. Thus, in this section, we will focus on studies in human cells.

Key studies have reported that depletion of KAP1, HP1 [163,210], or disruption of HP1 dimerization [210] promotes efficient DSB processing, likely by alleviating chromatin compaction barriers. However, there is strong evidence in human cells for a positive role of heterochromatin factors, particularly during early damage sensing and spatial organization of repair, whereas persistent retention can impede later repair steps.

##### HP1

All HP1 isoforms are recruited to DSBs [224] and their loss results in defective repair [225]. In particular, HP1γ interacts with the BARD1–BRCA1 complex, supporting its stable retention at DSBs [226,227]. Similarly, HP1ɑ is recruited to DSBs via the Human Chromatin Assembly Factor-1 (CAF-1) complex and promotes HR [228,229], potentially via phase-separation of recombinatorial domains, as described in a recent study [229]. This study also agrees with the previous literature [220] that HP1β is quickly dispersed from damage sites [229], highlighting differential roles of HP1 isoforms in DSB repair within heterochromatin. It is important to note some inconsistencies in studies of HP1 isoforms and pathway choice. For instance, studies have shown that HP1γ both inhibits [230] and promotes [225,227] HR and that HP1β both biases toward NHEJ [229] and promotes end resection [230]. Future work is needed to clarify the regulation of HP1 isoforms within heterochromatin (and potentially euchromatin) and their differential contribution to DSB repair. Notably, HP1ɑ and HP1β display heterochromatic localization while HP1γ is localized broadly across euchromatin and heterochromatin [231], pointing toward differential mechanisms between isoforms arising in different chromatin contexts.

##### H3K9me3

H3K9me3 is transiently deposited at DSB sites to activate the HAT, TIP60 [232], a key regulator of DSB sensing that acetylates and activates ATM and facilitates chromatin remodeling via the p400 ATPase complex [83,233,234]. This deposition requires a complex containing KAP1, HP1, and the methyltransferase SUV39H1, and this complex is quickly removed via ATM-dependent phosphorylation of KAP1 [232]. Importantly, this was observed in euchromatic-originating breaks, pointing to a role of transient heterochromatization in the repair of DSBs in an open chromatin context.

Together, these findings suggest that efficient DSB repair requires transient and spatially restricted recruitment of heterochromatin-associated factors, coupled with timely chromatin relaxation, rather than persistent compaction. The extent to which these mechanisms, namely HP1 isoform contributions and transient deposition of SUV39H1-complexes, occur differentially based on chromatin context requires more investigation.

### 3.3. Chromatin Re-Establishment

How and to what extent chromatin is reorganized to its native state after the extensive remodeling necessary for DSB repair is poorly understood. The best investigated mechanisms of chromatin restoration after the DSB response is through (1) the deposition of histone variants and their chaperones and (2) the removal of damage-associated marks [235,236].

The deposition of histone variants through the action of histone chaperones is the best characterized mechanism of chromatin restoration. Conceptually, the deposition of new histones near the break site is thought to replace the extensive disassembly of histones reported at the break site under damage [143]. In response to UVC damage, which mainly induces bulky DNA lesions repaired by nucleotide excision repair (NER), histone variants H3.1 and H2A.X have been shown to be deposited by chaperones CAF-1 and Facilitates Chromatin Transcription (FACT), respectively [237,238]. FACT has additionally been implicated in H2A/H2B deposition in response to UVC damage [239] and was found to play chromatin remodeling roles early in HR repair [240]. It seems likely that mechanisms of chromatin restoration partially overlap between NER and DSB repair, as evidenced by the observation that knockdown of CAF-1 blocks chromatin reassembly in DSBs as well [143]. In response to DSBs, H3.3 has been shown to be deposited by the Alpha-Thalassemia/Mental Retardation Syndrome X-linked and Death Domain Associated Protein (ATRX/DAXX) complex during HR repair synthesis [241], the chaperone Histone Cell Cycle Regulator (HIRA) to promote reassembly [143], and in a CHD2-dependent mechanism early in the DSB response to trigger chromatin expansion [242].

In human cells, phosphatases have been implicated in removing the damage γH2AX signal [243,244,245,246] and deubiquitinating enzymes (DUBs) in removing RNF8/RNF168-mediated histone ubiquitination [247,248,249]. Conversely, the deposition of H3K56ac signals chromatin reassembly after DSB repair in yeast [250], although its role in human DSB repair remains controversial, as previously discussed.

A recent study using a combination of quantitative imaging approaches revealed that transcriptional repression in response to a DSB occurs within TAD-defined regions in cuts up to 2.12 Mb away and results in alteration of the TAD structure, both of which are heritable after multiple cell divisions, suggesting that the 3D architecture of the genome is not fully restored after DSB repair [251]. This observation is mechanistically consistent with earlier findings that newly synthesized histones carry a restricted and distinct PTM signature prior to chromatin incorporation [252], raising the possibility that incomplete restoration of pre-existing chromatin states during DSB repair contributes to persistent transcriptional alterations. Further work will be required to investigate the extent to which this is relevant in human disease and whether DSB burden may accumulate through repeated epigenetically imperfect chromatin restoration events.

## 4. Conclusions 

Chromatin exerts multifaceted control over DSB repair by functioning as a scaffold for damage signaling and pathway choice, as well as a physical barrier. Local nucleosome organization and PTMs regulate immediate accessibility and recruitment of repair factors, whereas ATP-dependent remodelers mediate nucleosome sliding, eviction, and histone exchange critical for efficient DSB repair. Disruptions of these mechanisms by metabolic changes, disease-associated mutations, and genetic variation in chromatin regulators alter repair pathway choice and contribute to genomic instability and human disease. Continued integration of orthogonal and high-resolution approaches will be essential to resolve outstanding mechanistic questions, thus translating insights into therapeutic strategies that exploit chromatin context to modulate repair outcomes.

## Data Availability

No new data were created or analyzed in this study.

## References

[1] Kornberg R.D. (1974). Chromatin Structure: A Repeating Unit of Histones and DNA. Science.

[2] Luijsterburg M.S., van Attikum H. (2011). Chromatin and the DNA Damage Response: The Cancer Connection. Mol. Oncol..

[3] Demetriadou C., Koufaris C., Kirmizis A. (2020). Histone N-Alpha Terminal Modifications: Genome Regulation at the Tip of the Tail. Epigenet. Chromatin.

[4] Cedar H., Bergman Y. (2009). Linking DNA Methylation and Histone Modification: Patterns and Paradigms. Nat. Rev. Genet..

[5] Cuvier O., Fierz B. (2017). Dynamic Chromatin Technologies: From Individual Molecules to Epigenomic Regulation in Cells. Nat. Rev. Genet..

[6] Morrison O., Thakur J. (2021). Molecular Complexes at Euchromatin, Heterochromatin and Centromeric Chromatin. Int. J. Mol. Sci..

[7] Kouzarides T. (2007). Chromatin Modifications and Their Function. Cell.

[8] Clarke T.L., Mostoslavsky R. (2022). DNA Repair as a Shared Hallmark in Cancer and Ageing. Mol. Oncol..

[9] Noll M. (1974). Internal Structure of the Chromatin Subunit. Nucleic Acids Res..

[10] Hara R., Mo J., Sancar A. (2000). DNA Damage in the Nucleosome Core Is Refractory to Repair by Human Excision Nuclease. Mol. Cell. Biol..

[11] Tolstorukov M.Y., Volfovsky N., Stephens R.M., Park P.J. (2011). Impact of Chromatin Structure on Sequence Variability in the Human Genome. Nat. Struct. Mol. Biol..

[12] Brambilla F., Garcia-Manteiga J.M., Monteleone E., Hoelzen L., Zocchi A., Agresti A., Bianchi M.E. (2020). Nucleosomes Effectively Shield DNA from Radiation Damage in Living Cells. Nucleic Acids Res..

[13] Takata H., Hanafusa T., Mori T., Shimura M., Iida Y., Ishikawa K., Yoshikawa K., Yoshikawa Y., Maeshima K. (2013). Chromatin Compaction Protects Genomic DNA from Radiation Damage. PLoS ONE.

[14] Jackson S.P., Bartek J. (2009). The DNA-Damage Response in Human Biology and Disease. Nature.

[15] Caracciolo D., Riillo C., Di Martino M.T., Tagliaferri P., Tassone P. (2021). Alternative Non-Homologous End-Joining: Error-Prone DNA Repair as Cancer’s Achilles’ Heel. Cancers.

[16] Sfeir A., Symington L.S. (2015). Microhomology-Mediated End Joining: A Back-up Survival Mechanism or Dedicated Pathway?. Trends Biochem. Sci..

[17] Bhargava R., Onyango D.O., Stark J.M. (2016). Regulation of Single Strand Annealing and Its Role in Genome Maintenance. Trends Genet. TIG.

[18] Chang H.H.Y., Pannunzio N.R., Adachi N., Lieber M.R. (2017). Non-Homologous DNA End Joining and Alternative Pathways to Double-Strand Break Repair. Nat. Rev. Mol. Cell Biol..

[19] Walker J.R., Corpina R.A., Goldberg J. (2001). Structure of the Ku Heterodimer Bound to DNA and Its Implications for Double-Strand Break Repair. Nature.

[20] Scully R., Panday A., Elango R., Willis N.A. (2019). DNA Double-Strand Break Repair-Pathway Choice in Somatic Mammalian Cells. Nat. Rev. Mol. Cell Biol..

[21] Cannavo E., Cejka P. (2014). Sae2 Promotes dsDNA Endonuclease Activity within Mre11–Rad50–Xrs2 to Resect DNA Breaks. Nature.

[22] Sartori A.A., Lukas C., Coates J., Mistrik M., Fu S., Bartek J., Baer R., Lukas J., Jackson S.P. (2007). Human CtIP Promotes DNA End Resection. Nature.

[23] Nimonkar A.V., Genschel J., Kinoshita E., Polaczek P., Campbell J.L., Wyman C., Modrich P., Kowalczykowski S.C. (2011). BLM-DNA2-RPA-MRN and EXO1-BLM-RPA-MRN Constitute Two DNA End Resection Machineries for Human DNA Break Repair. Genes Dev..

[24] Jensen R.B., Carreira A., Kowalczykowski S.C. (2010). Purified Human BRCA2 Stimulates RAD51-Mediated Recombination. Nature.

[25] Maréchal A., Zou L. (2015). RPA-Coated Single-Stranded DNA as a Platform for Post-Translational Modifications in the DNA Damage Response. Cell Res..

[26] Sy S.M.H., Huen M.S.Y., Chen J. (2009). PALB2 Is an Integral Component of the BRCA Complex Required for Homologous Recombination Repair. Proc. Natl. Acad. Sci. USA.

[27] Zhang F., Ma J., Wu J., Ye L., Cai H., Xia B., Yu X. (2009). PALB2 Links BRCA1 and BRCA2 in the DNA-Damage Response. Curr. Biol..

[28] Filippo J.S., Sung P., Klein H. (2008). Mechanism of Eukaryotic Homologous Recombination. Annu. Rev. Biochem..

[29] Jasin M., Rothstein R. (2013). Repair of Strand Breaks by Homologous Recombination. Cold Spring Harb. Perspect. Biol..

[30] Smerdon M.J., Lieberman M.W. (1978). Nucleosome Rearrangement in Human Chromatin during UV-Induced DNA- Reapir Synthesis. Proc. Natl. Acad. Sci. USA.

[31] Smerdon M.J. (1991). DNA Repair and the Role of Chromatin Structure. Curr. Opin. Cell Biol..

[32] Gangaraju V.K., Bartholomew B. (2007). Mechanisms of ATP Dependent Chromatin Remodeling. Mutat. Res..

[33] Simon M., North J.A., Shimko J.C., Forties R.A., Ferdinand M.B., Manohar M., Zhang M., Fishel R., Ottesen J.J., Poirier M.G. (2011). Histone Fold Modifications Control Nucleosome Unwrapping and Disassembly. Proc. Natl. Acad. Sci. USA.

[34] Rogakou E.P., Pilch D.R., Orr A.H., Ivanova V.S., Bonner W.M. (1998). DNA Double-Stranded Breaks Induce Histone H2AX Phosphorylation on Serine 139. J. Biol. Chem..

[35] Falck J., Coates J., Jackson S.P. (2005). Conserved Modes of Recruitment of ATM, ATR and DNA-PKcs to Sites of DNA Damage. Nature.

[36] Stewart G.S., Wang B., Bignell C.R., Taylor A.M.R., Elledge S.J. (2003). MDC1 Is a Mediator of the Mammalian DNA Damage Checkpoint. Nature.

[37] Stucki M., Clapperton J.A., Mohammad D., Yaffe M.B., Smerdon S.J., Jackson S.P. (2005). MDC1 Directly Binds Phosphorylated Histone H2AX to Regulate Cellular Responses to DNA Double-Strand Breaks. Cell.

[38] de Jager M., van Noort J., van Gent D.C., Dekker C., Kanaar R., Wyman C. (2001). Human Rad50/Mre11 Is a Flexible Complex That Can Tether DNA Ends. Mol. Cell.

[39] Lee J.-H., Paull T.T. (2005). ATM Activation by DNA Double-Strand Breaks Through the Mre11-Rad50-Nbs1 Complex. Science.

[40] Duursma A.M., Driscoll R., Elias J.E., Cimprich K.A. (2013). A Role for the MRN Complex in ATR Activation via TOPBP1 Recruitment. Mol. Cell.

[41] Lou Z., Minter-Dykhouse K., Franco S., Gostissa M., Rivera M.A., Celeste A., Manis J.P., van Deursen J., Nussenzweig A., Paull T.T. (2006). MDC1 Maintains Genomic Stability by Participating in the Amplification of ATM-Dependent DNA Damage Signals. Mol. Cell.

[42] Kolas N.K., Chapman J.R., Nakada S., Ylanko J., Chahwan R., Sweeney F.D., Panier S., Mendez M., Wildenhain J., Thomson T.M. (2007). Orchestration of the DNA-Damage Response by the RNF8 Ubiquitin Ligase. Science.

[43] Reinhardt H.C., Yaffe M.B. (2013). Phospho-Ser/Thr-Binding Domains: Navigating the Cell Cycle and DNA Damage Response. Nat. Rev. Mol. Cell Biol..

[44] Doil C., Mailand N., Bekker-Jensen S., Menard P., Larsen D.H., Pepperkok R., Ellenberg J., Panier S., Durocher D., Bartek J. (2009). RNF168 Binds and Amplifies Ubiquitin Conjugates on Damaged Chromosomes to Allow Accumulation of Repair Proteins. Cell.

[45] Fradet-Turcotte A., Canny M.D., Escribano-Díaz C., Orthwein A., Leung C.C.Y., Huang H., Landry M.-C., Kitevski-LeBlanc J., Noordermeer S.M., Sicheri F. (2013). 53BP1 Is a Reader of the DNA-Damage-Induced H2A Lys 15 Ubiquitin Mark. Nature.

[46] Stewart G.S., Stankovic T., Byrd P.J., Wechsler T., Miller E.S., Huissoon A., Drayson M.T., West S.C., Elledge S.J., Taylor A.M.R. (2007). RIDDLE Immunodeficiency Syndrome Is Linked to Defects in 53BP1-Mediated DNA Damage Signaling. Proc. Natl. Acad. Sci. USA.

[47] Stewart G.S., Panier S., Townsend K., Al-Hakim A.K., Kolas N.K., Miller E.S., Nakada S., Ylanko J., Olivarius S., Mendez M. (2009). The RIDDLE Syndrome Protein Mediates a Ubiquitin-Dependent Signaling Cascade at Sites of DNA Damage. Cell.

[48] Riboldi G.M., Samanta D., Asuncion R.M.D., Frucht S. (2026). Ataxia-Telangiectasia. StatPearls.

[49] Zhu T., Zheng J.-Y., Huang L.-L., Wang Y.-H., Yao D.-F., Dai H.-B. (2023). Human PARP1 Substrates and Regulators of Its Catalytic Activity: An Updated Overview. Front. Pharmacol..

[50] Haince J.-F., McDonald D., Rodrigue A., Déry U., Masson J.-Y., Hendzel M.J., Poirier G.G. (2008). PARP1-Dependent Kinetics of Recruitment of MRE11 and NBS1 Proteins to Multiple DNA Damage Sites. J. Biol. Chem..

[51] Ray Chaudhuri A., Nussenzweig A. (2017). The Multifaceted Roles of PARP1 in DNA Repair and Chromatin Remodelling. Nat. Rev. Mol. Cell Biol..

[52] Price B.D., D’Andrea A.D. (2013). Chromatin Remodeling at DNA Double-Strand Breaks. Cell.

[53] Messner S., Altmeyer M., Zhao H., Pozivil A., Roschitzki B., Gehrig P., Rutishauser D., Huang D., Caflisch A., Hottiger M.O. (2010). PARP1 ADP-Ribosylates Lysine Residues of the Core Histone Tails. Nucleic Acids Res..

[54] Poirier G.G., de Murcia G., Jongstra-Bilen J., Niedergang C., Mandel P. (1982). Poly(ADP-Ribosyl)Ation of Polynucleosomes Causes Relaxation of Chromatin Structure. Proc. Natl. Acad. Sci. USA.

[55] Haince J.-F., Kozlov S., Dawson V.L., Dawson T.M., Hendzel M.J., Lavin M.F., Poirier G.G. (2007). Ataxia Telangiectasia Mutated (ATM) Signaling Network Is Modulated by a Novel Poly(ADP-Ribose)-Dependent Pathway in the Early Response to DNA-Damaging Agents. J. Biol. Chem..

[56] Dulev S., Tkach J., Lin S., Batada N.N. (2014). SET8 Methyltransferase Activity during the DNA Double-strand Break Response Is Required for Recruitment of 53BP1. EMBO Rep..

[57] Xu L., Zhang L., Sun J., Hu X., Kalvakolanu D.V., Ren H., Guo B. (2022). Roles for the Methyltransferase SETD8 in DNA Damage Repair. Clin. Epigenet..

[58] Wu H., Siarheyeva A., Zeng H., Lam R., Dong A., Wu X.-H., Li Y., Schapira M., Vedadi M., Min J. (2013). Crystal Structures of the Human Histone H4K20 Methyltransferases SUV420H1 and SUV420H2. FEBS Lett..

[59] Mallette F.A., Mattiroli F., Cui G., Young L.C., Hendzel M.J., Mer G., Sixma T.K., Richard S. (2012). RNF8- and RNF168-Dependent Degradation of KDM4A/JMJD2A Triggers 53BP1 Recruitment to DNA Damage Sites. EMBO J..

[60] Drané P., Brault M.-E., Cui G., Meghani K., Chaubey S., Detappe A., Parnandi N., He Y., Zheng X.-F., Botuyan M.V. (2017). TIRR Regulates 53BP1 by Masking Its Histone Methyl-Lysine Binding Function. Nature.

[61] Ye Q., Ma J., Wang Z., Li L., Liu T., Wang B., Zhu L., Lei Y., Xu S., Wang K. (2024). DTX3L-Mediated TIRR Nuclear Export and Degradation Regulates DNA Repair Pathway Choice and PARP Inhibitor Sensitivity. Nat. Commun..

[62] Pei H., Zhang L., Luo K., Qin Y., Chesi M., Fei F., Bergsagel P.L., Wang L., You Z., Lou Z. (2011). MMSET Regulates Histone H4K20 Methylation and 53BP1 Accumulation at DNA Damage Sites. Nature.

[63] Pellegrino S., Michelena J., Teloni F., Imhof R., Altmeyer M. (2017). Replication-Coupled Dilution of H4K20me2 Guides 53BP1 to Pre-Replicative Chromatin. Cell Rep..

[64] Oda H., Hübner M.R., Beck D.B., Vermeulen M., Hurwitz J., Spector D.L., Reinberg D. (2010). Regulation of the Histone H4 Monomethylase PR-Set7 by CRL4(Cdt2)-Mediated PCNA-Dependent Degradation during DNA Damage. Mol. Cell.

[65] Hernández-Reyes Y., Paz-Cabrera M.C., Freire R., Smits V.A.J., Hernández-Reyes Y., Paz-Cabrera M.C., Freire R., Smits V.A.J. (2022). USP29 Deubiquitinates SETD8 and Regulates DNA Damage-Induced H4K20 Monomethylation and 53BP1 Focus Formation. Cells.

[66] Perez Y., Alhourani F., Patouillard J., Ribeyre C., Larroque M., Baldin V., Lleres D., Grimaud C., Julien E. (2025). Cell-Cycle Dependent Inhibition of BRCA1 Signaling by the Lysine Methyltransferase SET8. Cell Cycle.

[67] Duro E., Lundin C., Ask K., Sanchez-Pulido L., MacArtney T.J., Toth R., Ponting C.P., Groth A., Helleday T., Rouse J. (2010). Identification of the MMS22L-TONSL Complex That Promotes Homologous Recombination. Mol. Cell.

[68] Saredi G., Huang H., Hammond C.M., Alabert C., Bekker-Jensen S., Forne I., Reverón-Gómez N., Foster B.M., Mlejnkova L., Bartke T. (2016). H4K20me0 Marks Post-Replicative Chromatin and Recruits the TONSL–MMS22L DNA Repair Complex. Nature.

[69] Nakamura K., Saredi G., Becker J.R., Foster B.M., Nguyen N.V., Beyer T.E., Cesa L.C., Faull P.A., Lukauskas S., Frimurer T. (2019). H4K20me0 Recognition by BRCA1–BARD1 Directs Homologous Recombination to Sister Chromatids. Nat. Cell Biol..

[70] Herviou L., Ovejero S., Izard F., Karmous-Gadacha O., Gourzones C., Bellanger C., De Smedt E., Ma A., Vincent L., Cartron G. (2021). Targeting the Methyltransferase SETD8 Impairs Tumor Cell Survival and Overcomes Drug Resistance Independently of P53 Status in Multiple Myeloma. Clin. Epigenet..

[71] Milite C., Feoli A., Viviano M., Rescigno D., Cianciulli A., Balzano A.L., Mai A., Castellano S., Sbardella G. (2016). The Emerging Role of Lysine Methyltransferase SETD8 in Human Diseases. Clin. Epigenet..

[72] Zhang X., Chen Z., He X., Wang J., Zhong J., Zou Y., Zheng X., Lin Y., Zhang R., Kang T. (2025). SUMOylation of SETD8 Promotes Tumor Growth by Methylating and Stabilizing MYC in Bladder Cancer. Adv. Sci..

[73] Wang X., Cao C., Tan X., Liao X., Du X., Wang X., Liu T., Gong D., Hu Z., Tian X. (2023). SETD8, a Frequently Mutated Gene in Cervical Cancer, Enhances Cisplatin Sensitivity by Impairing DNA Repair. Cell Biosci..

[74] Musselman C.A., Lalonde M.-E., Côté J., Kutateladze T.G. (2012). Perceiving the Epigenetic Landscape through Histone Readers. Nat. Struct. Mol. Biol..

[75] Li G., Reinberg D. (2011). Chromatin Higher-Order Structures and Gene Regulation. Curr. Opin. Genet. Dev..

[76] Shahbazian M.D., Grunstein M. (2007). Functions of Site-Specific Histone Acetylation and Deacetylation. Annu. Rev. Biochem..

[77] Steunou A.-L., Rossetto D., Côté J., Workman J.L., Abmayr S.M. (2014). Regulating Chromatin by Histone Acetylation. Fundamentals of Chromatin.

[78] Chiu L.-Y., Gong F., Miller K.M. (2017). Bromodomain Proteins: Repairing DNA Damage within Chromatin. Philos. Trans. R. Soc. B Biol. Sci..

[79] Gong F., Miller K.M. (2018). Double Duty: ZMYND8 in the DNA Damage Response and Cancer. Cell Cycle.

[80] Ogiwara H., Ui A., Otsuka A., Satoh H., Yokomi I., Nakajima S., Yasui A., Yokota J., Kohno T. (2011). Histone Acetylation by CBP and P300 at Double-Strand Break Sites Facilitates SWI/SNF Chromatin Remodeling and the Recruitment of Non-Homologous End Joining Factors. Oncogene.

[81] Billon P., Côté J. (2012). Precise Deposition of Histone H2A.Z in Chromatin for Genome Expression and Maintenance. Biochim. Biophys. Acta BBA-Gene Regul. Mech..

[82] Ikura T., Tashiro S., Kakino A., Shima H., Jacob N., Amunugama R., Yoder K., Izumi S., Kuraoka I., Tanaka K. (2007). DNA Damage-Dependent Acetylation and Ubiquitination of H2AX Enhances Chromatin Dynamics. Mol. Cell. Biol..

[83] Murr R., Loizou J.I., Yang Y.-G., Cuenin C., Li H., Wang Z.-Q., Herceg Z. (2006). Histone Acetylation by Trrap–Tip60 Modulates Loading of Repair Proteins and Repair of DNA Double-Strand Breaks. Nat. Cell Biol..

[84] Sun Y., Jiang X., Chen S., Fernandes N., Price B.D. (2005). A Role for the Tip60 Histone Acetyltransferase in the Acetylation and Activation of ATM. Proc. Natl. Acad. Sci. USA.

[85] Tang J., Cho N.W., Cui G., Manion E.M., Shanbhag N.M., Botuyan M.V., Mer G., Greenberg R.A. (2013). Acetylation Limits 53BP1 Association with Damaged Chromatin to Promote Homologous Recombination. Nat. Struct. Mol. Biol..

[86] Clarke T.L., Sanchez-Bailon M.P., Chiang K., Reynolds J.J., Herrero-Ruiz J., Bandeiras T.M., Matias P.M., Maslen S.L., Skehel J.M., Stewart G.S. (2017). PRMT5-Dependent Methylation of the TIP60 Coactivator RUVBL1 Is a Key Regulator of Homologous Recombination. Mol. Cell.

[87] Hamard P.-J., Santiago G.E., Liu F., Karl D.L., Martinez C., Man N., Mookhtiar A.K., Duffort S., Greenblatt S., Verdun R.E. (2018). PRMT5 Regulates DNA Repair by Controlling the Alternative Splicing of Histone-Modifying Enzymes. Cell Rep..

[88] Jacquet K., Fradet-Turcotte A., Avvakumov N., Lambert J.-P., Roques C., Pandita R.K., Paquet E., Herst P., Gingras A.-C., Pandita T.K. (2016). The TIP60 Complex Regulates Bivalent Chromatin Recognition by 53BP1 through Direct H4K20me Binding and H2AK15 Acetylation. Mol. Cell.

[89] Vempati R.K., Jayani R.S., Notani D., Sengupta A., Galande S., Haldar D. (2010). P300-Mediated Acetylation of Histone H3 Lysine 56 Functions in DNA Damage Response in Mammals. J. Biol. Chem..

[90] Das C., Lucia M.S., Hansen K.C., Tyler J.K. (2009). CBP/P300-Mediated Acetylation of Histone H3 on Lysine 56. Nature.

[91] Korotkov A., Seluanov A., Gorbunova V. (2021). Sirtuin 6: Linking Longevity with Genome and Epigenome Stability. Trends Cell Biol..

[92] Mostoslavsky R., Chua K.F., Lombard D.B., Pang W.W., Fischer M.R., Gellon L., Liu P., Mostoslavsky G., Franco S., Murphy M.M. (2006). Genomic Instability and Aging-like Phenotype in the Absence of Mammalian SIRT6. Cell.

[93] Sebastián C., Zwaans B.M.M., Silberman D.M., Gymrek M., Goren A., Zhong L., Ram O., Truelove J., Guimaraes A.R., Toiber D. (2012). The histone deacetylase SIRT6 is a tumor suppressor that controls cancer metabolism. Cell.

[94] Toiber D., Erdel F., Bouazoune K., Silberman D.M., Zhong L., Mulligan P., Sebastian C., Cosentino C., Martinez-Pastor B., Giacosa S. (2013). SIRT6 Recruits SNF2H to DNA Break Sites, Preventing Genomic Instability through Chromatin Remodeling. Mol. Cell.

[95] Stopka T., Skoultchi A.I. (2003). The ISWI ATPase Snf2h Is Required for Early Mouse Development. Proc. Natl. Acad. Sci. USA.

[96] White R.R., Vijg J. (2016). Do DNA Double-Strand Breaks Drive Aging?. Mol. Cell.

[97] Firsanov D., Zacher M., Tian X., Sformo T.L., Zhao Y., Tombline G., Lu J.Y., Zheng Z., Perelli L., Gurreri E. (2025). Evidence for Improved DNA Repair in Long-Lived Bowhead Whale. Nature.

[98] Tian X., Firsanov D., Zhang Z., Cheng Y., Luo L., Tombline G., Tan R., Simon M., Henderson S., Steffan J. (2019). SIRT6 Is Responsible for More Efficient DNA Double-Strand Break Repair in Long-Lived Species. Cell.

[99] Ferrer C.M., Alders M., Postma A.V., Park S., Klein M.A., Cetinbas M., Pajkrt E., Glas A., van Koningsbruggen S., Christoffels V.M. (2018). An Inactivating Mutation in the Histone Deacetylase SIRT6 Causes Human Perinatal Lethality. Genes Dev..

[100] Vadla R., Chatterjee N., Haldar D. (2020). Cellular Environment Controls the Dynamics of Histone H3 Lysine 56 Acetylation in Response to DNA Damage in Mammalian Cells. J. Biosci..

[101] Hargreaves D.C., Crabtree G.R. (2011). ATP-Dependent Chromatin Remodeling: Genetics, Genomics and Mechanisms. Cell Res..

[102] Reyes A.A., Marcum R.D., He Y. (2021). Structure and Function of ATP-Dependent Chromatin Remodeling Complexes. J. Mol. Biol..

[103] Vignali M., Hassan A.H., Neely K.E., Workman J.L. (2000). ATP-Dependent Chromatin-Remodeling Complexes. Mol. Cell. Biol..

[104] Cairns B.R., Lorch Y., Li Y., Zhang M., Lacomis L., Erdjument-Bromage H., Tempst P., Du J., Laurent B., Kornberg R.D. (1996). RSC, an Essential, Abundant Chromatin-Remodeling Complex. Cell.

[105] Côté J., Quinn J., Workman J.L., Peterson C.L. (1994). Stimulation of GAL4 Derivative Binding to Nucleosomal DNA by the Yeast SWI/SNF Complex. Science.

[106] Egel R., Beach D.H., Klar A.J. (1984). Genes Required for Initiation and Resolution Steps of Mating-Type Switching in Fission Yeast. Proc. Natl. Acad. Sci. USA.

[107] Neigeborn L., Carlson M. (1984). Genes Affecting the Regulation of SUC2 Gene Expression by Glucose Repression in Saccharomyces Cerevisiae. Genetics.

[108] Peterson C.L., Herskowitz I. (1992). Characterization of the Yeast SWI1, SWI2, and SWI3 Genes, Which Encode a Global Activator of Transcription. Cell.

[109] Chen K., Yuan J., Sia Y., Chen Z. (2023). Mechanism of Action of the SWI/SNF Family Complexes. Nucleus.

[110] Michel B.C., D’Avino A.R., Cassel S.H., Mashtalir N., McKenzie Z.M., McBride M.J., Valencia A.M., Zhou Q., Bocker M., Soares L.M.M. (2018). A Non-Canonical SWI/SNF Complex Is a Synthetic Lethal Target in Cancers Driven by BAF Complex Perturbation. Nat. Cell Biol..

[111] Centore R.C., Sandoval G.J., Soares L.M.M., Kadoch C., Chan H.M. (2020). Mammalian SWI/SNF Chromatin Remodeling Complexes: Emerging Mechanisms and Therapeutic Strategies. Trends Genet..

[112] Qi W., Wang R., Chen H., Wang X., Xiao T., Boldogh I., Ba X., Han L., Zeng X. (2015). BRG1 Promotes the Repair of DNA Double-Strand Breaks by Facilitating the Replacement of RPA with RAD51. J. Cell Sci..

[113] Vélez-Cruz R., Manickavinayaham S., Biswas A.K., Clary R.W., Premkumar T., Cole F., Johnson D.G. (2016). RB Localizes to DNA Double-Strand Breaks and Promotes DNA End Resection and Homologous Recombination through the Recruitment of BRG1. Genes Dev..

[114] Hays E., Nettleton E., Carter C., Morales M., Vo L., Passo M., Vélez-Cruz R. (2020). The SWI/SNF ATPase BRG1 Stimulates DNA End Resection and Homologous Recombination by Reducing Nucleosome Density at DNA Double Strand Breaks and by Promoting the Recruitment of the CtIP Nuclease. Cell Cycle.

[115] Chen Y., Zhang H., Xu Z., Tang H., Geng A., Cai B., Su T., Shi J., Jiang C., Tian X. (2019). A PARP1–BRG1–SIRT1 Axis Promotes HR Repair by Reducing Nucleosome Density at DNA Damage Sites. Nucleic Acids Res..

[116] Shen J., Peng Y., Wei L., Zhang W., Yang L., Lan L., Kapoor P., Ju Z., Mo Q., Shih I.-M. (2015). ARID1A Deficiency Impairs the DNA Damage Checkpoint and Sensitizes Cells to PARP Inhibitors. Cancer Discov..

[117] Bakr A., Della Corte G., Veselinov O., Kelekçi S., Chen M.-J.M., Lin Y.-Y., Sigismondo G., Iacovone M., Cross A., Syed R. (2024). ARID1A Regulates DNA Repair through Chromatin Organization and Its Deficiency Triggers DNA Damage-Mediated Anti-Tumor Immune Response. Nucleic Acids Res..

[118] Kanno S., Kobayashi T., Watanabe R., Kurimasa A., Tanaka K., Yasui A., Ui A. (2025). Armadillo Domain of ARID1A Directly Interacts with DNA-PKcs to Couple Chromatin Remodeling with Nonhomologous End Joining (NHEJ) Pathway. Nucleic Acids Res..

[119] Arnould C., Rocher V., Finoux A.-L., Clouaire T., Li K., Zhou F., Caron P., Mangeot P.E., Ricci E.P., Mourad R. (2021). Loop Extrusion as a Mechanism for Formation of DNA Damage Repair Foci. Nature.

[120] Fudenberg G., Imakaev M., Lu C., Goloborodko A., Abdennur N., Mirny L.A. (2016). Formation of Chromosomal Domains by Loop Extrusion. Cell Rep..

[121] Zhu G., Liu J., Li Y., Huang H., Chen C., Wu D., Cao P., Su L., Wang Y., Zhang H. (2024). ARID1B Deficiency Leads to Impaired DNA Damage Response and Activated cGAS-STING Pathway in Non-Small Cell Lung Cancer. J. Cancer.

[122] Zernickel E., Sak A., Riaz A., Klein D., Groneberg M., Stuschke M. (2019). Targeting of BRM Sensitizes BRG1-Mutant Lung Cancer Cell Lines to Radiotherapy. Mol. Cancer Ther..

[123] Caumanns J.J., Wisman G.B.A., Berns K., van der Zee A.G.J., de Jong S. (2018). *ARID1A* Mutant Ovarian Clear Cell Carcinoma: A Clear Target for Synthetic Lethal Strategies. Biochim. Biophys. Acta BBA-Rev. Cancer.

[124] Davidson J., Shen Z., Gong X., Pollack J.R. (2017). SWI/SNF Aberrations Sensitize Pancreatic Cancer Cells to DNA Crosslinking Agents. Oncotarget.

[125] Watanabe R., Ui A., Kanno S., Ogiwara H., Nagase T., Kohno T., Yasui A. (2014). SWI/SNF Factors Required for Cellular Resistance to DNA Damage Include ARID1A and ARID1B and Show Interdependent Protein Stability. Cancer Res..

[126] Erkek S., Johann P.D., Finetti M.A., Drosos Y., Chou H.-C., Zapatka M., Sturm D., Jones D.T.W., Korshunov A., Rhyzova M. (2019). Comprehensive Analysis of Chromatin States in Atypical Teratoid/Rhabdoid Tumor Identifies Diverging Roles for SWI/SNF and Polycomb in Gene Regulation. Cancer Cell.

[127] Beddow R.A., Smith M., Kidd A., Corbett R., Hunter A.G. (2011). Diagnosis of Distal 22q11.2 Deletion Syndrome in a Patient with a Teratoid/Rhabdoid Tumour. Eur. J. Med. Genet..

[128] Ben-Shachar S., Ou Z., Shaw C.A., Belmont J.W., Patel M.S., Hummel M., Amato S., Tartaglia N., Berg J., Sutton V.R. (2008). 22q11.2 Distal Deletion: A Recurrent Genomic Disorder Distinct from DiGeorge Syndrome and Velocardiofacial Syndrome. Am. J. Hum. Genet..

[129] Del Baldo G., Carta R., Alessi I., Merli P., Agolini E., Rinelli M., Boccuto L., Milano G.M., Serra A., Carai A. (2021). Rhabdoid Tumor Predisposition Syndrome: From Clinical Suspicion to General Management. Front. Oncol..

[130] Clarke T.L., Cho H.M., Ceppi I., Gao B., Yadav T., Silveira G.G., Boon R., Martinez-Pastor B., Amoh N.Y.A., Machin B. (2025). ZNF280A Links DNA Double-Strand Break Repair to Human 22q11.2 Distal Deletion Syndrome. Nat. Cell Biol..

[131] Poli J., Gasser S.M., Papamichos-Chronakis M. (2017). The INO80 Remodeller in Transcription, Replication and Repair. Philos. Trans. R. Soc. B Biol. Sci..

[132] Gerhold C.B., Winkler D.D., Lakomek K., Seifert F.U., Fenn S., Kessler B., Witte G., Luger K., Hopfner K.-P. (2012). Structure of Actin-Related Protein 8 and Its Contribution to Nucleosome Binding. Nucleic Acids Res..

[133] Osakabe A., Takahashi Y., Murakami H., Otawa K., Tachiwana H., Oma Y., Nishijima H., Shibahara K., Kurumizaka H., Harata M. (2014). DNA Binding Properties of the Actin-Related Protein Arp8 and Its Role in DNA Repair. PLoS ONE.

[134] Cai Y., Jin J., Yao T., Gottschalk A.J., Swanson S.K., Wu S., Shi Y., Washburn M.P., Florens L., Conaway R.C. (2007). YY1 Functions with INO80 to Activate Transcription. Nat. Struct. Mol. Biol..

[135] Chen L., Cai Y., Jin J., Florens L., Swanson S.K., Washburn M.P., Conaway J.W., Conaway R.C. (2011). Subunit Organization of the Human INO80 Chromatin Remodeling Complex: An Evolutionarily Conserved Core Complex Catalyzes ATP-Dependent Nucleosome Remodeling. J. Biol. Chem..

[136] Peng Q., Wan D., Zhou R., Luo H., Wang J., Ren L., Zeng Y., Yu C., Zhang S., Huang X. (2022). The Biological Function of Metazoan-Specific Subunit Nuclear Factor Related to kappaB Binding Protein of INO80 Complex. Int. J. Biol. Macromol..

[137] Kashiwaba S., Kitahashi K., Watanabe T., Onoda F., Ohtsu M., Murakami Y. (2010). The Mammalian INO80 Complex Is Recruited to DNA Damage Sites in an ARP8 Dependent Manner. Biochem. Biophys. Res. Commun..

[138] Morrison A.J., Highland J., Krogan N.J., Arbel-Eden A., Greenblatt J.F., Haber J.E., Shen X. (2004). INO80 and γ-H2AX Interaction Links ATP-Dependent Chromatin Remodeling to DNA Damage Repair. Cell.

[139] Gospodinov A., Vaissiere T., Krastev D.B., Legube G., Anachkova B., Herceg Z. (2011). Mammalian Ino80 Mediates Double-Strand Break Repair through Its Role in DNA End Strand Resection. Mol. Cell. Biol..

[140] Alatwi H.E., Downs J.A. (2015). Removal of H2A.Z by INO80 Promotes Homologous Recombination. EMBO Rep..

[141] Xu Y., Ayrapetov M.K., Xu C., Gursoy-Yuzugullu O., Hu Y., Price B.D. (2012). Histone H2A.Z Controls a Critical Chromatin Remodeling Step Required for DNA Double-Strand Break Repair. Mol. Cell.

[142] Keil J.M., Doyle D.Z., Qalieh A., Lam M.M., Funk O.H., Qalieh Y., Shi L., Mohan N., Sorel A., Kwan K.Y. (2020). Symmetric Neural Progenitor Divisions Require Chromatin-Mediated Homologous Recombination DNA Repair by Ino80. Nat. Commun..

[143] Li X., Tyler J.K. (2016). Nucleosome Disassembly during Human Non-Homologous End Joining Followed by Concerted HIRA- and CAF-1-Dependent Reassembly. eLife.

[144] Saravanan M., Wuerges J., Bose D., McCormack E.A., Cook N.J., Zhang X., Wigley D.B. (2012). Interactions between the Nucleosome Histone Core and Arp8 in the INO80 Chromatin Remodeling Complex. Proc. Natl. Acad. Sci. USA.

[145] Cullati S.N., Akizuki K., Shan Y., Zhang E., Ren L., Guillen R.X., Turner L.A., Chen J.-S., Navarrete-Perea J., Elmore Z.C. (2024). The DNA Damage Repair Function of Fission Yeast CK1 Involves Targeting Arp8, a Subunit of the INO80 Chromatin Remodeling Complex. Mol. Cell. Biol..

[146] Lee S.-A., Lee H.-S., Hur S.-K., Kang S.W., Oh G.T., Lee D., Kwon J. (2017). INO80 Haploinsufficiency Inhibits Colon Cancer Tumorigenesis via Replication Stress-Induced Apoptosis. Oncotarget.

[147] Zhang S., Zhou B., Wang L., Li P., Bennett B., Snyder R., Garantziotis S., Fargo D., Cox A.D., Chen L. (2017). INO80 Is Required for Oncogenic Transcription and Tumor Growth in Non-Small Cell Lung Cancer. Oncogene.

[148] Lim H., Kwon O., La H., Lee H., Lee H., Do J.-T., Song H., Choi Y., Hong K. (2026). INO80 Regulates Promoter-Associated R-Loops to Coordinate Transcription and Maintain Genome Stability in Embryonic Stem Cells. Biol. Res..

[149] García-Muse T., Aguilera A. (2019). R Loops: From Physiological to Pathological Roles. Cell.

[150] Prendergast L., McClurg U.L., Hristova R., Berlinguer-Palmini R., Greener S., Veitch K., Hernandez I., Pasero P., Rico D., Higgins J.M.G. (2020). Resolution of R-Loops by INO80 Promotes DNA Replication and Maintains Cancer Cell Proliferation and Viability. Nat. Commun..

[151] Aydin Ö.Z., Vermeulen W., Lans H. (2014). ISWI Chromatin Remodeling Complexes in the DNA Damage Response. Cell Cycle.

[152] Lan L., Ui A., Nakajima S., Hatakeyama K., Hoshi M., Watanabe R., Janicki S.M., Ogiwara H., Kohno T., Kanno S. (2010). The ACF1 Complex Is Required for DNA Double-Strand Break Repair in Human Cells. Mol. Cell.

[153] Nakamura K., Kato A., Kobayashi J., Yanagihara H., Sakamoto S., Oliveira D.V.N.P., Shimada M., Tauchi H., Suzuki H., Tashiro S. (2011). Regulation of Homologous Recombination by RNF20-Dependent H2B Ubiquitination. Mol. Cell.

[154] Atsumi Y., Minakawa Y., Ono M., Dobashi S., Shinohe K., Shinohara A., Takeda S., Takagi M., Takamatsu N., Nakagama H. (2015). ATM and SIRT6/SNF2H Mediate Transient H2AX Stabilization When DSBs Form by Blocking HUWE1 to Allow Efficient γH2AX Foci Formation. Cell Rep..

[155] Smeenk G., Wiegant W.W., Marteijn J.A., Luijsterburg M.S., Sroczynski N., Costelloe T., Romeijn R.J., Pastink A., Mailand N., Vermeulen W. (2013). Poly(ADP-Ribosyl)Ation Links the Chromatin Remodeler SMARCA5/SNF2H to RNF168-Dependent DNA Damage Signaling. J. Cell Sci..

[156] Vidi P.-A., Liu J., Salles D., Jayaraman S., Dorfman G., Gray M., Abad P., Moghe P.V., Irudayaraj J.M., Wiesmüller L. (2014). NuMA Promotes Homologous Recombination Repair by Regulating the Accumulation of the ISWI ATPase SNF2h at DNA Breaks. Nucleic Acids Res..

[157] Min S., Jo S., Lee H.-S., Chae S., Lee J.-S., Ji J.-H., Cho H. (2014). ATM-Dependent Chromatin Remodeler Rsf-1 Facilitates DNA Damage Checkpoints and Homologous Recombination Repair. Cell Cycle.

[158] Min S., Lee H.-S., Ji J.-H., Heo Y., Kim Y., Chae S., Choi Y.W., Kang H.-C., Nakanishi M., Cho H. (2021). The Chromatin Remodeler RSF1 Coordinates Epigenetic Marks for Transcriptional Repression and DSB Repair. Nucleic Acids Res..

[159] Li Y., Gong H., Wang P., Zhu Y., Peng H., Cui Y., Li H., Liu J., Wang Z. (2021). The Emerging Role of ISWI Chromatin Remodeling Complexes in Cancer. J. Exp. Clin. Cancer Res..

[160] Längst G., Manelyte L. (2015). Chromatin Remodelers: From Function to Dysfunction. Genes.

[161] Sims J.K., Wade P.A. (2011). SnapShot: Chromatin Remodeling: CHD. Cell.

[162] Stanley F.K.T., Moore S., Goodarzi A.A. (2013). CHD Chromatin Remodelling Enzymes and the DNA Damage Response. Mutat. Res.-Fundam. Mol. Mech. Mutagen..

[163] Goodarzi A.A., Noon A.T., Deckbar D., Ziv Y., Shiloh Y., Löbrich M., Jeggo P.A. (2008). ATM Signaling Facilitates Repair of DNA Double-Strand Breaks Associated with Heterochromatin. Mol. Cell.

[164] Goodarzi A.A., Kurka T., Jeggo P.A. (2011). KAP-1 Phosphorylation Regulates CHD3 Nucleosome Remodeling during the DNA Double-Strand Break Response. Nat. Struct. Mol. Biol..

[165] Noon A.T., Shibata A., Rief N., Löbrich M., Stewart G.S., Jeggo P.A., Goodarzi A.A. (2010). 53BP1-Dependent Robust Localized KAP-1 Phosphorylation Is Essential for Heterochromatic DNA Double-Strand Break Repair. Nat. Cell Biol..

[166] Ziv Y., Bielopolski D., Galanty Y., Lukas C., Taya Y., Schultz D.C., Lukas J., Bekker-Jensen S., Bartek J., Shiloh Y. (2006). Chromatin Relaxation in Response to DNA Double-Strand Breaks Is Modulated by a Novel ATM- and KAP-1 Dependent Pathway. Nat. Cell Biol..

[167] Chou D.M., Adamson B., Dephoure N.E., Tan X., Nottke A.C., Hurov K.E., Gygi S.P., Colaiácovo M.P., Elledge S.J. (2010). A Chromatin Localization Screen Reveals Poly (ADP Ribose)-Regulated Recruitment of the Repressive Polycomb and NuRD Complexes to Sites of DNA Damage. Proc. Natl. Acad. Sci. USA.

[168] Larsen D.H., Poinsignon C., Gudjonsson T., Dinant C., Payne M.R., Hari F.J., Rendtlew Danielsen J.M., Menard P., Sand J.C., Stucki M. (2010). The Chromatin-Remodeling Factor CHD4 Coordinates Signaling and Repair after DNA Damage. J. Cell Biol..

[169] Polo S.E., Kaidi A., Baskcomb L., Galanty Y., Jackson S.P. (2010). Regulation of DNA-Damage Responses and Cell-Cycle Progression by the Chromatin Remodelling Factor CHD4. EMBO J..

[170] Smeenk G., Wiegant W.W., Vrolijk H., Solari A.P., Pastink A., van Attikum H. (2010). The NuRD Chromatin–Remodeling Complex Regulates Signaling and Repair of DNA Damage. J. Cell Biol..

[171] Pan M.-R., Hsieh H.-J., Dai H., Hung W.-C., Li K., Peng G., Lin S.-Y. (2012). Chromodomain Helicase DNA-Binding Protein 4 (CHD4) Regulates Homologous Recombination DNA Repair, and Its Deficiency Sensitizes Cells to Poly(ADP-Ribose) Polymerase (PARP) Inhibitor Treatment. J. Biol. Chem..

[172] Hou T., Cao Z., Zhang J., Tang M., Tian Y., Li Y., Lu X., Chen Y., Wang H., Wei F.-Z. (2020). SIRT6 Coordinates with CHD4 to Promote Chromatin Relaxation and DNA Repair. Nucleic Acids Res..

[173] Kolla V., Zhuang T., Higashi M., Naraparaju K., Brodeur G.M. (2014). Role of CHD5 in Human Cancers: 10 Years Later. Cancer Res..

[174] Kolla V., Naraparaju K., Zhuang T., Higashi M., Kolla S., Blobel G.A., Brodeur G.M. (2015). The Tumour Suppressor CHD5 Forms a NuRD-Type Chromatin Remodelling Complex. Biochem. J..

[175] Potts R.C., Zhang P., Wurster A.L., Precht P., Mughal M.R., Iii W.H.W., Zhang Y., Becker K.G., Mattson M.P., Pazin M.J. (2011). CHD5, a Brain-Specific Paralog of Mi2 Chromatin Remodeling Enzymes, Regulates Expression of Neuronal Genes. PLoS ONE.

[176] Hall W.A., Petrova A.V., Colbert L.E., Hardy C.W., Fisher S.B., Saka B., Shelton J.W., Warren M.D., Pantazides B.G., Gandhi K. (2014). Low CHD5 Expression Activates the DNA Damage Response and Predicts Poor Outcome in Patients Undergoing Adjuvant Therapy for Resected Pancreatic Cancer. Oncogene.

[177] Spruijt C.G., Luijsterburg M.S., Menafra R., Lindeboom R.G.H., Jansen P.W.T.C., Edupuganti R.R., Baltissen M.P., Wiegant W.W., Voelker-Albert M.C., Matarese F. (2016). ZMYND8 Co-Localizes with NuRD on Target Genes and Regulates Poly(ADP-Ribose)-Dependent Recruitment of GATAD2A/NuRD to Sites of DNA Damage. Cell Rep..

[178] Liu Z., Ajit K., Wu Y., Zhu W.-G., Gullerova M. (2024). The GATAD2B-NuRD Complex Drives DNA:RNA Hybrid-Dependent Chromatin Boundary Formation upon DNA Damage. EMBO J..

[179] Hoffmann A., Spengler D. (2019). Chromatin Remodeling Complex NuRD in Neurodevelopment and Neurodevelopmental Disorders. Front. Genet..

[180] Pierson T.M., Otero M.G., Grand K., Choi A., Graham J.M., Young J.I., Mackay J.P. (2019). The NuRD Complex and Macrocephaly Associated Neurodevelopmental Disorders. Am. J. Med. Genet. Part C Semin. Med. Genet..

[181] Lai A.Y., Wade P.A. (2011). Cancer Biology and NuRD: A Multifaceted Chromatin Remodelling Complex. Nat. Rev. Cancer.

[182] Giles K.A., Taberlay P.C., Cesare A.J., Jones M.J.K. (2025). Roles for the 3D Genome in the Cell Cycle, DNA Replication, and Double Strand Break Repair. Front. Cell Dev. Biol..

[183] Aten J.A., Stap J., Krawczyk P.M., van Oven C.H., Hoebe R.A., Essers J., Kanaar R. (2004). Dynamics of DNA Double-Strand Breaks Revealed by Clustering of Damaged Chromosome Domains. Science.

[184] Aymard F., Aguirrebengoa M., Guillou E., Javierre B.M., Bugler B., Arnould C., Rocher V., Iacovoni J.S., Biernacka A., Skrzypczak M. (2017). Genome-Wide Mapping of Long-Range Contacts Unveils Clustering of DNA Double-Strand Breaks at Damaged Active Genes. Nat. Struct. Mol. Biol..

[185] Arnould C., Rocher V., Saur F., Bader A.S., Muzzopappa F., Collins S., Lesage E., Le Bozec B., Puget N., Clouaire T. (2023). Chromatin Compartmentalization Regulates the Response to DNA Damage. Nature.

[186] Schrank B.R., Aparicio T., Li Y., Chang W., Chait B.T., Gundersen G.G., Gottesman M.E., Gautier J. (2018). Nuclear ARP2/3 Drives DNA Break Clustering for Homology-Directed Repair. Nature.

[187] Zagelbaum J., Schooley A., Zhao J., Schrank B.R., Callen E., Zha S., Gottesman M.E., Nussenzweig A., Rabadan R., Dekker J. (2022). Multiscale Reorganization of the Genome Following DNA Damage Facilitates Chromosome Translocations via Nuclear Actin Polymerization. Nat. Struct. Mol. Biol..

[188] Collins P.L., Purman C., Porter S.I., Nganga V., Saini A., Hayer K.E., Gurewitz G.L., Sleckman B.P., Bednarski J.J., Bassing C.H. (2020). DNA Double-Strand Breaks Induce H2Ax Phosphorylation Domains in a Contact-Dependent Manner. Nat. Commun..

[189] Mehta S., Zhang J. (2022). Liquid–Liquid Phase Separation Drives Cellular Function and Dysfunction in Cancer. Nat. Rev. Cancer.

[190] Kilic S., Lezaja A., Gatti M., Bianco E., Michelena J., Imhof R., Altmeyer M. (2019). Phase Separation of 53BP1 Determines Liquid-like Behavior of DNA Repair Compartments. EMBO J..

[191] Levone B.R., Lenzken S.C., Antonaci M., Maiser A., Rapp A., Conte F., Reber S., Mechtersheimer J., Ronchi A.E., Mühlemann O. (2021). FUS-Dependent Liquid-Liquid Phase Separation Is Important for DNA Repair Initiation. J. Cell Biol..

[192] Pessina F., Giavazzi F., Yin Y., Gioia U., Vitelli V., Galbiati A., Barozzi S., Garre M., Oldani A., Flaus A. (2019). Functional Transcription Promoters at DNA Double-Strand Breaks Mediate RNA-Driven Phase Separation of Damage-Response Factors. Nat. Cell Biol..

[193] Miné-Hattab J., Liu S., Taddei A., Miné-Hattab J., Liu S., Taddei A. (2022). Repair Foci as Liquid Phase Separation: Evidence and Limitations. Genes.

[194] Chen J., Zhang W., Ma Y., Yan X., Wang Y., Ouyang Q., Wu M., Yang G. (2025). Temporal and Spatial Dynamics of DNA Double-Strand Break Repair Centers. DNA Repair.

[195] Fedkenheuer M., Shang Y., Jung S., Fedkenheuer K., Park S., Mazza D., Sebastian R., Nagashima H., Zong D., Tan H. (2025). A Dual Role of Cohesin in DNA DSB Repair. Nat. Commun..

[196] Kim B.-J., Li Y., Zhang J., Xi Y., Li Y., Yang T., Jung S.Y., Pan X., Chen R., Li W. (2010). Genome-Wide Reinforcement of Cohesin Binding at Pre-Existing Cohesin Sites in Response to Ionizing Radiation in Human Cells. J. Biol. Chem..

[197] Kim S.-T., Xu B., Kastan M.B. (2002). Involvement of the Cohesin Protein, Smc1, in Atm-Dependent and Independent Responses to DNA Damage. Genes Dev..

[198] Caron P., Aymard F., Iacovoni J.S., Briois S., Canitrot Y., Bugler B., Massip L., Losada A., Legube G. (2012). Cohesin Protects Genes against γH2AX Induced by DNA Double-Strand Breaks. PLoS Genet..

[199] Sanders J.T., Freeman T.F., Xu Y., Golloshi R., Stallard M.A., Hill A.M., San Martin R., Balajee A.S., McCord R.P. (2020). Radiation-Induced DNA Damage and Repair Effects on 3D Genome Organization. Nat. Commun..

[200] Hu J. (2025). A Tale of Two Forms of Cohesin in DNA Repair. Science.

[201] Marin-Gonzalez A., Rybczynski A.T., Nilavar N.M., Nguyen D., Li A.G., Karwacki-Neisius V., Zou R.S., Avilés-Vázquez F.J., Kanemaki M.T., Scully R. (2025). Cohesin Drives Chromatin Scanning during the RAD51-Mediated Homology Search. Science.

[202] Teloni F., Takacs Z., Mitter M., Langer C.C.H., Prlesi I., Steinacker T.L., Reuter V.P., Mylarshchikov D., Gerlich D.W. (2025). Cohesin Guides Homology Search during DNA Repair Using Loops and Sister Chromatid Linkages. Science.

[203] Kovatcheva M., Liao W., Klein M.E., Robine N., Geiger H., Crago A.M., Dickson M.A., Tap W.D., Singer S., Koff A. (2017). ATRX Is a Regulator of Therapy Induced Senescence in Human Cells. Nat. Commun..

[204] Loe T.K., Li J.S.Z., Zhang Y., Azeroglu B., Boddy M.N., Denchi E.L. (2020). Telomere Length Heterogeneity in ALT Cells Is Maintained by PML-Dependent Localization of the BTR Complex to Telomeres. Genes Dev..

[205] Węsierska-Gądek J., Schloffer D., Kotala V., Horky M. (2002). Escape of P53 Protein from E6-Mediated Degradation in HeLa Cells after Cisplatin Therapy. Int. J. Cancer.

[206] Volpe E., Colantoni A., Corda L., Di Tommaso E., Pelliccia F., Ottalevi R., Guarracino A., Licastro D., Faino L., Capulli M. (2025). The Reference Genome of the Human Diploid Cell Line RPE-1. Nat. Commun..

[207] Monteiro A.N.A. (2003). BRCA1: The Enigma of Tissue-Specific Tumor Development. Trends Genet..

[208] Allshire R.C., Madhani H.D. (2018). Ten Principles of Heterochromatin Formation and Function. Nat. Rev. Mol. Cell Biol..

[209] Chiolo I., Altmeyer M., Legube G., Mekhail K. (2025). Nuclear and Genome Dynamics Underlying DNA Double-Strand Break Repair. Nat. Rev. Mol. Cell Biol..

[210] Mitrentsi I., Lou J., Kerjouan A., Verigos J., Reina-San-Martin B., Hinde E., Soutoglou E. (2022). Heterochromatic Repeat Clustering Imposes a Physical Barrier on Homologous Recombination to Prevent Chromosomal Translocations. Mol. Cell.

[211] Schuster-Böckler B., Lehner B. (2012). Chromatin Organization Is a Major Influence on Regional Mutation Rates in Human Cancer Cells. Nature.

[212] Zheng C.L., Wang N.J., Chung J., Moslehi H., Sanborn J.Z., Hur J.S., Collisson E.A., Vemula S.S., Naujokas A., Chiotti K.E. (2014). Transcription Restores DNA Repair to Heterochromatin, Determining Regional Mutation Rates in Cancer Genomes. Cell Rep..

[213] Yilmaz D., Furst A., Meaburn K., Lezaja A., Wen Y., Altmeyer M., Reina-San-Martin B., Soutoglou E. (2021). Activation of Homologous Recombination in G1 Preserves Centromeric Integrity. Nature.

[214] Saayman X., Graham E., Nathan W.J., Nussenzweig A., Esashi F. (2023). Centromeres as Universal Hotspots of DNA Breakage, Driving RAD51-Mediated Recombination during Quiescence. Mol. Cell.

[215] Zeitlin S.G., Baker N.M., Chapados B.R., Soutoglou E., Wang J.Y.J., Berns M.W., Cleveland D.W. (2009). Double-Strand DNA Breaks Recruit the Centromeric Histone CENP-A. Proc. Natl. Acad. Sci. USA.

[216] Shanbhag N.M., Rafalska-Metcalf I.U., Balane-Bolivar C., Janicki S.M., Greenberg R.A. (2010). ATM-Dependent Chromatin Changes Silence Transcription In *Cis* to DNA Double-Strand Breaks. Cell.

[217] Müller I., Merk B., Voss K.-O., Averbeck N., Jakob B., Durante M., Taucher-Scholz G. (2013). Species Conserved DNA Damage Response at the Inactive Human X Chromosome. Mutat. Res. Toxicol. Environ. Mutagen..

[218] Wensveen M.R., Dixit A.A., van Schendel R., Kendek A., Lambooij J.-P., Tijsterman M., Colmenares S.U., Janssen A. (2024). Double-Strand Breaks in Facultative Heterochromatin Require Specific Movements and Chromatin Changes for Efficient Repair. Nat. Commun..

[219] Kendek A., Wensveen M.R., Janssen A. (2021). The Sound of Silence: How Silenced Chromatin Orchestrates the Repair of Double-Strand Breaks. Genes.

[220] Ayoub N., Jeyasekharan A.D., Bernal J.A., Venkitaraman A.R. (2008). HP1-β Mobilization Promotes Chromatin Changes That Initiate the DNA Damage Response. Nature.

[221] Chiolo I., Minoda A., Colmenares S.U., Polyzos A., Costes S.V., Karpen G.H. (2011). Double-Strand Breaks in Heterochromatin Move Outside of a Dynamic HP1a Domain to Complete Recombinational Repair. Cell.

[222] Kendek A., Sandron A., Lambooij J.-P., Colmenares S.U., Pociunaite S.M., Gooijers I., de Groot L., Karpen G.H., Janssen A. (2024). DNA Double-Strand Break Movement in Heterochromatin Depends on the Histone Acetyltransferase dGcn5. Nucleic Acids Res..

[223] Ryu T., Spatola B., Delabaere L., Bowlin K., Hopp H., Kunitake R., Karpen G.H., Chiolo I. (2015). Heterochromatic Breaks Move to the Nuclear Periphery to Continue Recombinational Repair. Nat. Cell Biol..

[224] Luijsterburg M.S., Dinant C., Lans H., Stap J., Wiernasz E., Lagerwerf S., Warmerdam D.O., Lindh M., Brink M.C., Dobrucki J.W. (2009). Heterochromatin Protein 1 Is Recruited to Various Types of DNA Damage. J. Cell Biol..

[225] Lee Y.-H., Kuo C.-Y., Stark J.M., Shih H.-M., Ann D.K. (2013). HP1 Promotes Tumor Suppressor BRCA1 Functions during the DNA Damage Response. Nucleic Acids Res..

[226] Heath J., Cheyou E.S., Findlay S., Luo V.M., Carpio E.P., Lee J., Djerir B., Chen X., Morin T., Lebeau B. (2022). POGZ Promotes Homology-directed DNA Repair in an HP1-dependent Manner. EMBO Rep..

[227] Wu W., Nishikawa H., Fukuda T., Vittal V., Asano M., Miyoshi Y., Klevit R.E., Ohta T. (2015). Interaction of BARD1 and HP1 Is Required for BRCA1 Retention at Sites of DNA Damage. Cancer Res..

[228] Baldeyron C., Soria G., Roche D., Cook A.J.L., Almouzni G. (2011). HP1α Recruitment to DNA Damage by p150CAF-1 Promotes Homologous Recombination Repair. J. Cell Biol..

[229] Bohra D., Mazumder A. (2026). HP1 Isoforms Direct Repair Pathway Choice in Response to Heterochromatin Double-Strand Breaks. J. Cell Biol..

[230] Soria G., Almouzni G. (2013). Differential Contribution of HP1 Proteins to DNA End Resection and Homology-Directed Repair. Cell Cycle.

[231] Jones D.O., Cowell I.G., Singh P.B. (2000). Mammalian Chromodomain Proteins: Their Role in Genome Organisation and Expression. BioEssays.

[232] Ayrapetov M.K., Gursoy-Yuzugullu O., Xu C., Xu Y., Price B.D. (2014). DNA Double-Strand Breaks Promote Methylation of Histone H3 on Lysine 9 and Transient Formation of Repressive Chromatin. Proc. Natl. Acad. Sci. USA.

[233] Sun Y., Xu Y., Roy K., Price B.D. (2007). DNA Damage-Induced Acetylation of Lysine 3016 of ATM Activates ATM Kinase Activity. Mol. Cell. Biol..

[234] Xu Y., Sun Y., Jiang X., Ayrapetov M.K., Moskwa P., Yang S., Weinstock D.M., Price B.D. (2010). The P400 ATPase Regulates Nucleosome Stability and Chromatin Ubiquitination during DNA Repair. J. Cell Biol..

[235] Ferrand J., Rondinelli B., Polo S.E., Ferrand J., Rondinelli B., Polo S.E. (2020). Histone Variants: Guardians of Genome Integrity. Cells.

[236] Rossetto D., Truman A.W., Kron S.J., Côté J. (2010). Epigenetic Modifications in Double Strand Break DNA Damage Signaling and Repair. Clin. Cancer Res. Off. J. Am. Assoc. Cancer Res..

[237] Piquet S., Parc F.L., Bai S.-K., Chevallier O., Adam S., Polo S.E. (2018). The Histone Chaperone FACT Coordinates H2A.X-Dependent Signaling and Repair of DNA Damage. Mol. Cell.

[238] Polo S.E., Roche D., Almouzni G. (2006). New Histone Incorporation Marks Sites of UV Repair in Human Cells. Cell.

[239] Dinant C., Ampatziadis-Michailidis G., Lans H., Tresini M., Lagarou A., Grosbart M., Theil A.F., van Cappellen W.A., Kimura H., Bartek J. (2013). Enhanced Chromatin Dynamics by FACT Promotes Transcriptional Restart after UV-Induced DNA Damage. Mol. Cell.

[240] Oliveira D.V., Kato A., Nakamura K., Ikura T., Okada M., Kobayashi J., Yanagihara H., Saito Y., Tauchi H., Komatsu K. (2014). Histone Chaperone FACT Regulates Homologous Recombination by Chromatin Remodeling through Interaction with RNF20. J. Cell Sci..

[241] Juhász S., Elbakry A., Mathes A., Löbrich M. (2018). ATRX Promotes DNA Repair Synthesis and Sister Chromatid Exchange during Homologous Recombination. Mol. Cell.

[242] Luijsterburg M.S., de Krijger I., Wiegant W.W., Shah R.G., Smeenk G., de Groot A.J.L., Pines A., Vertegaal A.C.O., Jacobs J.J.L., Shah G.M. (2016). PARP1 Links CHD2-Mediated Chromatin Expansion and H3.3 Deposition to DNA Repair by Non-Homologous End-Joining. Mol. Cell.

[243] Cha H., Lowe J.M., Li H., Lee J.-S., Belova G.I., Bulavin D.V., Fornace A.J. (2010). Wip1 Directly Dephosphorylates γ-H2AX and Attenuates the DNA Damage Response. Cancer Res..

[244] Chowdhury D., Keogh M.-C., Ishii H., Peterson C.L., Buratowski S., Lieberman J. (2005). γ-H2AX Dephosphorylation by Protein Phosphatase 2A Facilitates DNA Double-Strand Break Repair. Mol. Cell.

[245] Macůrek L., Lindqvist A., Voets O., Kool J., Vos H.R., Medema R.H. (2010). Wip1 Phosphatase Is Associated with Chromatin and Dephosphorylates gammaH2AX to Promote Checkpoint Inhibition. Oncogene.

[246] Nakada S., Chen G.I., Gingras A.-C., Durocher D. (2008). PP4 Is a Gamma H2AX Phosphatase Required for Recovery from the DNA Damage Checkpoint. EMBO Rep..

[247] Mosbech A., Lukas C., Bekker-Jensen S., Mailand N. (2013). The Deubiquitylating Enzyme USP44 Counteracts the DNA Double-Strand Break Response Mediated by the RNF8 and RNF168 Ubiquitin Ligases. J. Biol. Chem..

[248] Shao G., Lilli D.R., Patterson-Fortin J., Coleman K.A., Morrissey D.E., Greenberg R.A. (2009). The Rap80-BRCC36 de-Ubiquitinating Enzyme Complex Antagonizes RNF8-Ubc13-Dependent Ubiquitination Events at DNA Double Strand Breaks. Proc. Natl. Acad. Sci. USA.

[249] Sharma N., Zhu Q., Wani G., He J., Wang Q., Wani A.A. (2014). USP3 Counteracts RNF168 via Deubiquitinating H2A and γH2AX at Lysine 13 and 15. Cell Cycle.

[250] Chen C.-C., Carson J.J., Feser J., Tamburini B., Zabaronick S., Linger J., Tyler J.K. (2008). Acetylated Lysine 56 on Histone H3 Drives Chromatin Assembly after Repair and Signals for the Completion of Repair. Cell.

[251] Bantele S., Mordini I., Biran A., Alcaraz N., Zonderland G., Wenger A., Krietenstein N., Groth A., Lukas J. (2025). Repair of DNA Double-Strand Breaks Leaves Heritable Impairment to Genome Function. Science.

[252] Loyola A., Bonaldi T., Roche D., Imhof A., Almouzni G. (2006). PTMs on H3 Variants before Chromatin Assembly Potentiate Their Final Epigenetic State. Mol. Cell.

